# Altered Expression of Glial Gap Junction Proteins Cx43, Cx30, and Cx47 in the 5XFAD Model of Alzheimer’s Disease

**DOI:** 10.3389/fnins.2020.582934

**Published:** 2020-10-07

**Authors:** Stella Angeli, Ioanna Kousiappa, Marios Stavrou, Irene Sargiannidou, Elena Georgiou, Savvas S. Papacostas, Kleopas A. Kleopa

**Affiliations:** ^1^Neurobiology Department, The Cyprus Institute of Neurology and Genetics, Nicosia, Cyprus; ^2^Cyprus School of Molecular Medicine, Nicosia, Cyprus; ^3^Department of Electrical and Computer Engineering, Faculty of Engineering, University of Cyprus, Nicosia, Cyprus; ^4^Neuroscience Department, The Cyprus Institute of Neurology and Genetics, Nicosia, Cyprus; ^5^Dementia and Cognitive Disorders Center, The Cyprus Institute of Neurology and Genetics, Nicosia, Cyprus; ^6^Medical School, University of Nicosia, Nicosia, Cyprus; ^7^Center for Neuromuscular disorders, The Cyprus Institute of Neurology and Genetics, Nicosia, Cyprus; ^8^Center for Multiple Sclerosis and Related Disorders, The Cyprus Institute of Neurology and Genetics, Nicosia, Cyprus

**Keywords:** Alzheimer’s disease, gap junctions, Cx43, Cx30, Cx47

## Abstract

Glial gap junction proteins, called connexins (Cxs), form gap junctions in the central nervous system (CNS) to allow the bidirectional cytosolic exchange of molecules between adjacent cells. Their involvement in inheritable diseases and the use of experimental animal models that closely mimic such diseases revealed the critical role of glial GJs in myelination and homeostasis. Cxs are also implicated in acquired demyelinating disorders, such as Multiple Sclerosis (MS) and Alzheimer’s disease (AD). Animal and human studies have revealed a role of the astrocytic Cx43 in the progression of AD but the role of Cx47, which is the main partner of Cx43 in the astrocyte-oligodendrocyte GJs is still unknown. The aim of this study was to investigate the astrocytic connexins, Cx43 and Cx30 in relation to oligodendrocytic Cx47 in the cortex and thalamus of the 5XFAD mouse model of AD. The model was characterized by increased Aβ deposition, gliosis, neuronal loss, and memory impairment. Compared to wild-type mice, Cx43 and Cx30 showed increased immunoreactivity in older 5XFAD mice, reflecting astrogliosis, while Cx47 immunoreactivity was reduced. Moreover, Cx47 GJ plaques showed reduced colocalization with Cx43 plaques. Oligodendrocyte precursor cells (OPCs) and mature oligodendrocyte populations were also depleted, and myelin deficits were observed. Our findings indicate reduced astrocyte-oligodendrocyte gap junction connectivity and possibly a shift in Cx43 expression toward astrocyte-astrocyte gap junctions and/or hemichannels, that could impair oligodendrocyte homeostasis and myelination. However, other factors, such as Aβ toxicity, could directly affect oligodendrocyte survival in AD. Our study provides evidence that Cxs might have implications in the progression of AD, although the role of oligodendrocyte Cxs in AD requires further investigation.

## Introduction

Alzheimer’s disease (AD) is the most common form of irreversible dementia and is characterized by the loss of cognitive function (Skaper, 2012). The hallmarks of AD include extracellular plaques formed by aggregated amyloid-β (Aβ) peptides and intracellular neurofibrillary tangles (aggregates of hyperphosphorylated tau protein). Familial AD mutations in the *APP* and *PSEN1* genes affect the proteolytic processing of the amyloid precursor protein (APP), with increased accumulation of Aβ peptides (especially Aβ_42_) causing neuronal toxicity (Hardy and Selkoe, 2002; Haass and Selkoe, 2007; Reiss et al., 2018). Tissue damage caused by Aβ accumulation was confirmed in 5XFAD mice, a transgenic mouse model of familial AD, by showing elevated levels of nitrotyrosine (NT), a toxic product of superoxide reaction with nitric oxide (NO) (Modi et al., 2015). Also, in AD, the inducible NO synthase (iNOS), which is produced by astrocytes, microglia and macrophages (Nathan et al., 2005) generates increased levels of NO which causes oxidative stress, consequently leading to neurodegeneration (Balez and Ooi, 2016). However, *INOS* gene expression levels in 5XFAD and control mice were found to be similar, indicating that iNOS is not excessively produced by glial cells of this mouse model (Griñán-Ferré et al., 2016).

In neurodegenerative diseases, like AD, glial cells, including astrocytes, microglia, and oligodendrocytes, activate and trigger hazardous neuroinflammatory reactions inducing neuronal death (Cragnolini et al., 2020). Glial cells are part of an organized network that offers communication and support via gap junctions (GJs) (Giaume et al., 2010). Adjacent glial cells form GJs which are composed of opposing hexamers of connexins (Cxs) (Yamasaki, 2018), which are involved in the exchange of small molecules and ions such as Ca^2+^, cAMP, ATP and glutamate and contribute to long distance intercellular signaling (Takeuchi and Suzumura, 2014). In the central nervous system (CNS), the astroglial-astroglial (A/A), and astroglial-oligodendroglial (A/O) coupling plays a major role in the maintenance of myelination, neuronal homeostasis, and synaptic neurotransmission (Yamasaki, 2018). Astrocytic Cx43 only couples with itself in the A/A GJs and with Cx47 in the A/O GJs, whereas astrocytic Cx30 couples with itself in the A/A GJs and with Cx32 in the A/O GJs (Nagy et al., 2003; Kleopa et al., 2004; Orthmann-Murphy et al., 2007).

Cxs are known to be implicated in disease either by mutations in Cx genes that cause inheritable diseases or by primary or secondary Cx dysfunctions that cause acquired disorders (Yamasaki, 2018). *GJB1* (Cx32) gene mutations cause the X-linked Charcot-Marie-Tooth disease, a disease of the peripheral nervous system, where Schwann cells fail to provide metabolic or trophic support for the physiological function of axons (Jeng et al., 2006). Autosomal recessive *GJC2* (Cx47) gene mutations cause the Pelizaeus Merzbacher-like disease, a hypomyelinating leukodystrophy (Uhlenberg et al., 2004). Also, oculodentodigital dysplasia is an autosomal dominant syndrome caused by *GJA1* (Cx43) gene mutations characterized by CNS demyelination and progressive spastic paraplegia (Paznekas et al., 2003), highlighting the importance of astrocytic coupling with oligodendrocytes, the myelinating cells of the CNS, for the homeostasis and survival of the latter. The crucial role of GJs for CNS myelination is validated in mouse models of these disorders (Sutor et al., 2000; Odermatt et al., 2003; Lutz et al., 2009; Sargiannidou et al., 2009; Magnotti et al., 2011; Tress et al., 2012). In addition, in mutant Superoxide dismutase-1 transgenic mice (mouse model of motor neuron disease), neuronal Cx36, which is responsible for the development of electrical synapses (Bautista et al., 2014), showed reduced expression (Belousov et al., 2018; Kobayakawa et al., 2018). In addition, the upregulation of Cx hemichannels (HCs) is linked to the activation of microglia and astrocytes in these mice (Cui et al., 2014). Cxs are also implicated in Multiple Sclerosis (MS) and the experimental autoimmune encephalomyelitis (EAE) mouse model of MS (Brand-Schieber et al., 2005; Eugenin et al., 2012; Markoullis et al., 2012a,b, 2014). In both MS and EAE, Cx32 was reduced within and around lesions, in acute and chronic stages, particularly in areas with severe inflammation (Markoullis et al., 2012a,b). In acute EAE, Cx47 was not reduced but rather redistributed from GJ plaques intracellularly, while in chronic EAE, Cx47 GJs were lost. However, Cx43 GJ plaques were disrupted in acute MS (Masaki, 2013) and EAE lesions (Markoullis et al., 2012a) but upregulated in chronic lesions, reflecting astrogliosis. Reactive astrocytes in MS chronic lesions were mostly coupled with other astrocytes forming Cx43-Cx43 GJs and formed less Cx43-Cx47 GJs with oligodendrocytes (Markoullis et al., 2012b).

In AD, astrocytic Cx43 and Cx30 expression was increased in AD post-mortem brain samples and in the APP/PS1 mouse model of AD, specifically around Aβ plaques (Nagy et al., 1996; Mei et al., 2010). Aβ peptide increases the propagation of [Ca]_i_^2+^ waves in astrocytes (Haughey and Mattson, 2003), a process in which Cxs are known to be involved (Scemes and Giaume, 2006). Low concentrations of monomerized Aβ_1__–__40_ impair gap junction communication in cultured rat astrocytes (Cruz et al., 2010). Aβ-induced HC opening was reported in astrocytic, microglial, and neuronal cultures obtained from transgenic mice (Orellana et al., 2011b). Results showed that Cx43 and Pannexin 1 (Panx1) HCs are involved in microglia, while only Cx43 HCs are activated in astrocytes. Cx43 and Panx1 HCs were shown to be activated mostly in reactive astrocytes infiltrating Aβ plaques, contributing to neuronal damage, in APP/PS1 mice (Yi et al., 2016). Aβ_2__5–3__5_-induced HC activation leads to ATP and glutamate release from microglia and astrocytes, triggering HC opening in neurons with detrimental consequences in neuronal survival (Orellana et al., 2011b). Also, it was reported that in culture models, Aβ not only triggers HC activation but also the activation of glial NMDA and P2X receptors, which in turn release ATP and glutamate, which then activate HC opening in neurons, leading to neuronal death (Koulakoff et al., 2012). Cx43 HC-mediated ATP release was reported in the APP/PS1 mouse model to further propagate the neurodegenerative process (Delekate et al., 2014). However, the implication of Cx47 in AD has not been investigated.

In the present report, we focused on the investigation of the astrocytic Cx43 and Cx30 and the main partner of Cx43 in the A/O GJs, Cx47, for the first time in the 5XFAD mouse model of AD. We show increased immunoreactivity of Cx43 and Cx30 at the microenvironment of Aβ plaques by immunostaining analysis, suggesting a role for Cx43 in the progression of the disease, also confirmed by the analysis of brain protein extracts in 5XFAD mice. Furthermore, we demonstrate decreased immunoreactivity of Cx47, for the first time in a mouse model of AD, indicating disruption of A/O GJs, together with depletion in oligodendrocyte precursor cells (OPCs) and mature oligodendrocytes, possibly contributing to neuronal degeneration and AD progression.

## Materials and Methods

### Experimental Mice

5XFAD mice overexpress high levels of mutant human APP (Swedish: K670N/M671L, Florida: I716V, London: V717I) and PSEN1 mutations (M146L, L286V mutations) under the presence of mouse Thy1 promoter, leading to rapid development of Aβ pathology and increased production of Aβ_42_ (Oakley et al., 2006). The 5XFAD transgenic mouse model of AD was purchased from Jackson Laboratory. Male 5XFAD mice were crossed with female SJL/B6 F1 mice and the offspring was used for all experiments. The offspring was divided in three age groups (3, 6 and 9 months of age, designated below as 3M, 6M, and 9M groups, respectively) and both transgenic and age matched wild-type (WT) mice were used. Animals were maintained in pathogen-free (SPF) conditions, in a 12 h light/dark cycle and were always provided with food and water. Animal care, sacrifice and experimental protocols used in this project follow the EU guidelines (Council Directive 86/609/EEC) and were approved by the Cyprus Government’s Veterinary Services (CY/EXP/PR.L1/2018).

### T-Maze Spontaneous Alternation Test

The T-maze test is a behavioral test which quantifies cognitive deficits in mice by testing their spatial memory and exploratory behavior (Deacon and Rawlins, 2006). It is an enclosed wooden apparatus which consists of the start alley (30 cm length × 10 cm width), two goal arms (30 cm length × 10 cm width), two guillotine doors which fit the maze and a central partition extending 7 cm into the start arm. The walls are 20 cm high. A thin layer of bedding (70% new and 30% old) was placed on the maze floor. The two guillotine doors were opened halfway, and the central partition was in place. Mice were placed in the start arm facing the wall and allowed to explore the start area. The selection of the goal arm (Left or Right) was recorded (all four appendages should enter the arm). As soon as the mouse had entered an arm, the guillotine door was closed, and the mouse was left in the arm for 30 s. The central partition was then removed from the apparatus. The mouse was gently removed from the arm and placed again in the start arm. The selection of the goal arm (Left or Right) was recorded again. Finally, the mice were given a score after completing a trial (0 = entry in the same arm, 1 = entry in the opposite arm (alternation), 2 = no entry/entry exceeding the 90 s time limit). By repeating trials, mice should have higher tendency to enter the not previously visited arm. All mice completed 20 trials (4 trials per day) and the percentage of spontaneous alternation was calculated.

### Histology

Mice were anesthetized intraperitoneally with Avertin and then transcardially perfused with normal saline followed by 4% paraformaldehyde (PFA). Half cerebrums were harvested, post-fixed with 4% PFA and cryopreserved in 20% sucrose in phosphate buffer (PB 0.1M) overnight. Optimum cutting temperature (OCT) compound was then used as the tissue embedding medium. The embedded cerebrums were then placed in a dry-ice/acetone cooling bath and stored in −80°C. Twelve μm-thick cryostat coronal sections were then obtained and stored in −20°C.

### Avidin-Biotin Complex (ABC) Immunohistochemistry

Coronal brain hemi-sections were permeabilized in cold acetone for 10 min, underwent quenching of endogenous peroxidase with 3% H_2_O_2_ in methanol for 30 min and pre-treated with 70% formic acid (antigen retrieval) for 5 min. Sections were then blocked at room temperature for 20min, with blocking solution [0.02% normal horse serum (NHS) in PBS/Triton X-100 (0.5%)], and incubated overnight at 4°C with anti-β-amyloid primary antibody (monoclonal 6E10, 1:400; Covance). Sections were then washed with PBS and incubated for 1 h with biotinylated secondary antibody which was detected with an avidin-biotin-peroxidase complex (Vectastain Elite ABC kit, Vector Labs, PK-6102) and 3,3′-Diaminobenzidine (DAB, DAKO, K3468). The sections were then counterstained with hematoxylin and mounted with glycerol.

### Fluorescence Immunohistochemistry

Sections were permeabilized in cold acetone for 10 min, blocked at room temperature with blocking solution [5% bovine serum albumin (BSA) in PBS/Triton X-100 (0.5%)] and incubated overnight at 4°C with the following mouse monoclonal primary antibodies: anti-β-amyloid (6E10, 1:400; Covance), anti-glial fibrillary acidic protein (GFAP, Sigma, 1:400), anti-APC (CC-1, Millipore, 1:50), anti-NeuN (Millipore, 1:400), anti-Cx47 (Invitrogen, 1:200), the following rabbit primary antibodies: anti- IBA1 (Biocare Medical, 1:500), anti-Cx43 (Cell Signaling, 1:50), anti-Cx30 (Thermo Fisher Scientific, 1:500), anti-Cx47 (Thermo Fisher Scientific, 1:500), anti-Cx32 (Thermo Fisher Scientific, 1:100) and anti-Olig2 (Millipore, 1:500) and the rat anti-PLP primary antibody (Prof. Reynold’s lab, 1:10). Sections were then washed with PBS and incubated for 1 h with the respective secondary antibodies: Fluorescein (FITC) goat anti-mouse, 1:100, Alexa Fluor® 594 goat anti-rabbit, 1:500, and rhodamine (TRITC) conjugated AffiniPure F (ab′)_2_ goat anti-rat, 1:2,000 (Jackson ImmunoResearch), stained with 4′,6′-diamidino-2-phenylindole (DAPI, Sigma-Aldrich) and mounted with fluorescent mounting medium (DAKO). Images were visualized by a Zeiss fluorescent microscope. To assess the astroglial and microglial activation, we measured the total area of GFAP and Iba1 immunofluorescence as a percentage of the total image area using the Image J software. NeuN^+^ neuronal numbers, Olig2^+^ cells (oligodendrocyte precursors and mature oligodendrocytes) and CC1^+^ cells (mature oligodendrocytes) were counted per total area in NeuN and Olig2/CC1 stained sections, respectively.

### Semi-Quantification of Fluorescence Intensity of Cx43, Cx30, and Cx47 at the Sites of Aβ Plaques

In 3- and 9-months-old 5XFAD mice, Aβ/Cx43 and Aβ/Cx30 immunostaining of brain sections were used to measure the fluorescence intensity of Cx43 and Cx30 at the level of plaques. Multiple images of the cortex and thalamus were captured with a 20x objective using a Leica Fluorescence Microscope (*n* = 6 mice per age). Aβ plaques found close to blood vessels were not considered. The total number of Aβ plaques measured were: 353 plaques in 3M cortex, 472 plaques in 3M thalamus, 740 plaques in 9M cortex and 1101 plaques in 9M thalamus. Image J software was used to analyze these images. For each Aβ plaque, three oval selections (with 60° rotation from each other) were made to cover the whole perimeter of the plaque and slightly outside of that perimeter. The mean fluorescence intensity of Cx43 and Cx30 was measured from the three oval selections at the level of each Aβ plaque and compared with the Cx fluorescence intensity in five areas away from Aβ plaques within the same image and significant differences (*p* < 0.05) between these values were identified by using the unpaired *t*-test (GraphPad Prism Software). From these measurements we were able to identify the percentage of Aβ plaques which had increased, decreased or unchanged Cx immunoreactivity compared to control areas away from Aβ plaques (Supplementary Figure 2). Also, the mean Cx fluorescence intensity at the level of Aβ plaques in 5XFAD mice was compared with the fluorescence intensity in areas away from Aβ plaques and with the Cx fluorescence intensity in WT mice (five different areas were measured per image). One-way ANOVA followed by Kruskal-Wallis test was used to detect any statistical significance between all age groups and the two genotypes.

Cx47 fluorescence intensity was measured in double immunostaining images for Aβ/Cx47 in 3- and 9-months-old 5XFAD mice and compared with WT controls. Oligodendrocytes (*n* = 31/image) with Cx47-positive puncta were selected with 6.858 mm^2^ squares and the fluorescence intensity was measured after removing the background. The statistical analysis was performed by the unpaired *t*-test.

### RNA Extraction and Quantitative Real-Time PCR

Cortex and thalamus were isolated from half cerebrums and RNA was extracted using the RNeasy lipid tissue mini kit (Qiagen). Tissues were homogenized with QIAzol lysis reagent and proteins were denatured with chloroform. DNase digestion was also performed, and total RNA was quantified using NanoDrop ND_100. TaqMan reverse transcription (RT) reagents were used for RT-PCR (final vol. 40 μl) in all samples (25°C for 10 min, 48°C for 30 min, and 95°C for 5 min). Also, quantitative real-time PCR was performed in all cDNAs to measure the expression of (*Gja1* (Cx43), *GJb6* (Cx30), *Gjc2* (Cx47), and *Gjb1* (Cx32) genes compared to the expression of *Tubb4a* (Tubulin, house-keeping gene) using the following Taqman Gene Expression assays: Cx43: Mm01179639_m1, Cx30: Mm00433661_s1, Cx47: Mm00519131-s1, Cx32: Mm01950058-s1, and Tubulin: Mm00726185_s1. A 7900HT Real-Time PCR System (Applied Biosystems, hold at 55°C for 2 min and at 95°C for 10 min, followed by 40 cycles at 95°C for 15 s and at 60°C for 1 min) was used for this purpose. Triplicates from each cDNA sample were loaded (200 ng) along with 1 μl of Taqman Gene Expression assay and 10 μl of Taqman Gene Expression Master mix in a final volume of 20 μl. Cycle thresholds (Cts) of genes of interest were normalized against tubulin and mRNA levels were calculated in 5XFAD and WT mice and shown as fold induction values (2^–ΔΔ*Ct*^) compared to WT control mice.

### Immunoblot Analysis

Mice were sacrificed and the cortex and thalamus were harvested from half cerebrums. The tissues were homogenized in ice-cold RIPA buffer (1% NP-40, 0.5% C_2__4_H_3__9_Na0_4_, 0.1%SDS, 2 mM EDTA) dissolved in 1xPBS along with a protease inhibitor cocktail (Roche) and centrifuged for 20 min at 4°C. Protein concentration was measured with bicinchoninic acid (BCA) assay (Smith et al., 1985) and 100 μg were mixed with 4x laemli buffer (Biorad). Tissue lysates were loaded in 12% SDS-PAGE [ddH_2_0, 30% acrylamide-bis (29:1), 1.5M Tris-CL pH 8.8, 10%SDS, 10%APS, Temed] and transferred to a Hybond PVDF blotting membrane (Life Sciences) using a semi-dry unit. The membrane was blocked in blocking solution [5% non-fat skimmed milk in PBS containing 0.1% Tween-20 (PBS-T)] for 1 h at room temperature and then incubated overnight at 4°C with the following mouse primary antibodies: anti-Cx43 (Millipore, 1:1,000) and anti-Tubulin-E7 (DSHB, 1:4,000) and the rabbit anti-Cx30 (Thermo Fisher Scientific, 1:500). After 15 min washes in PBS-T, membranes were incubated for 1 h at room temperature with a goat anti-mouse or anti-rabbit HRP-conjugated secondary antibody (Jackson Immunoresearch, 1:3,000). The bound antibody was visualized by enhanced chemiluminescence system (ECL, GE Healthcare Life Sciences), protein expression was normalized against tubulin and band intensity was quantified using Image J.

### Statistical Analysis

All data in graphs are expressed as the mean and error bars indicate the standard error of the mean (SEM). Normal distribution of data was evaluated, and the statistical tests performed were the following: One-way ANOVA followed by Sidak’s or Kruskal-Wallis multiple comparisons test and unpaired *t*-test. Values of *p* < 0.05 were considered significant. Details showing which statistical test was performed in each experiment are reported in the figure legends.

## Results

### Amyloid-Beta Pathology in 5XFAD Mice

**FIGURE 1:**
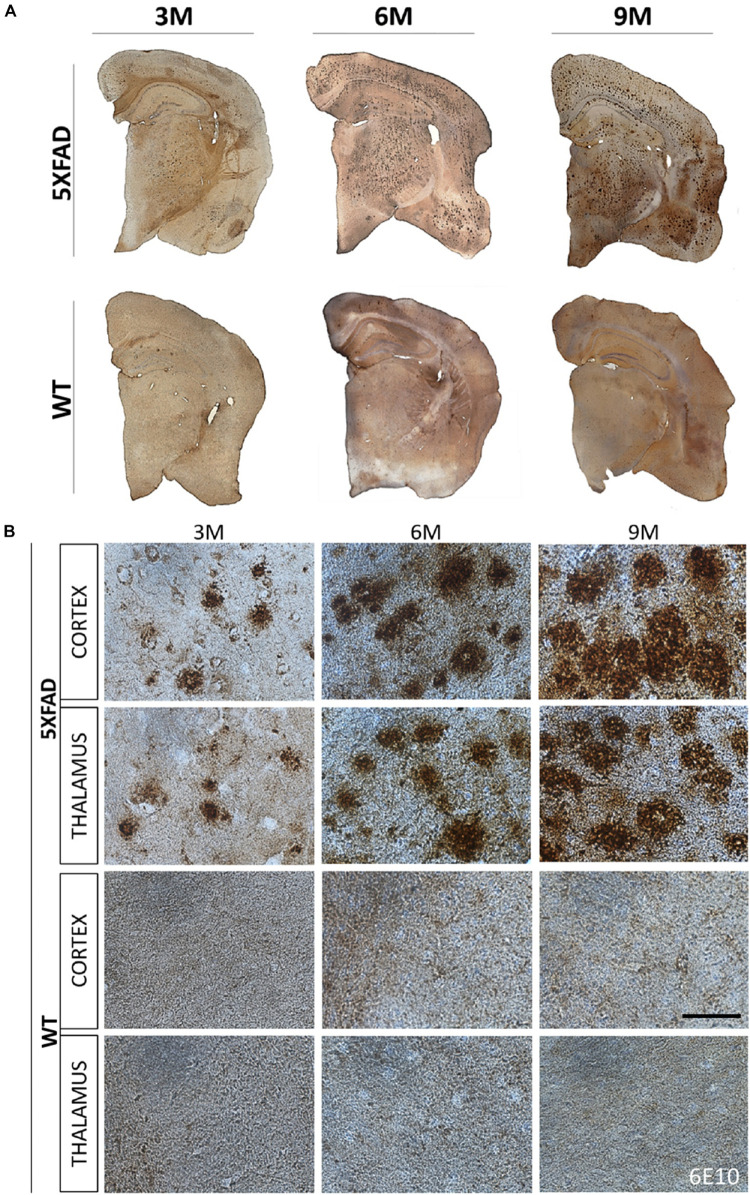
Progressive formation of Aβ plaques in 5XFAD mice. Brain DAB immunohistochemistry showed Aβ deposition in three age groups of 5XFAD mice (3, 6, and 9 months). **(A)** Coronal brain hemi-sections (12 μm thick) were stained with 6E10 primary antibody followed by DAB chromogen to reveal individual plaques (brown dots) in the cortex, thalamus and hippocampus. **(B)** Aβ plaque deposition increased tremendously with age as shown in higher magnification images of 5XFAD as opposed to WT mice in both brain areas. Scale bar = 50 μm in **(B)**.

The localization of Aβ deposits was investigated at 3, 6, and 9 months of age. DAB immunohistochemistry was performed in 5XFAD mice as well as in their age-matched WT littermate controls. Aβ accumulation in 5XFAD mice has been shown to progress with age (Oakley et al., 2006). Likewise, we observed in 3- months-old 5XFAD mice a few Aβ deposits in the cortex (layer VI) and in the thalamus. Aβ deposition gradually increased with age and larger deposits were observed in 9- months-old 5XFAD mice compared to 3- and 6-months-old animals (Figures 1A,B). Plaques at 6 and 9 months of age covered all cortical layers and the whole retrosplenial area of the cortex but were found most prominently in the layers V and VI of all other cortical areas (posterior parietal, primary somatosensory, auditory, temporal, ectorhinal, perirhinal, and entorhinal area). By 9 months of age the larger sized plaques were localized in cortical layer V. Moreover, at 6 and 9 months of age Aβ deposits were also found in the hippocampus mainly in the stratum oriens of the CA1 area and in the molecular and polymorph layers of the dentate gyrus. They also covered the whole thalamic area. WT mice showed no deposits in all ages.

### Neuronal Loss and Hippocampal Impairment in 6- and 9-Months-Old 5XFAD Mice

**FIGURE 2:**
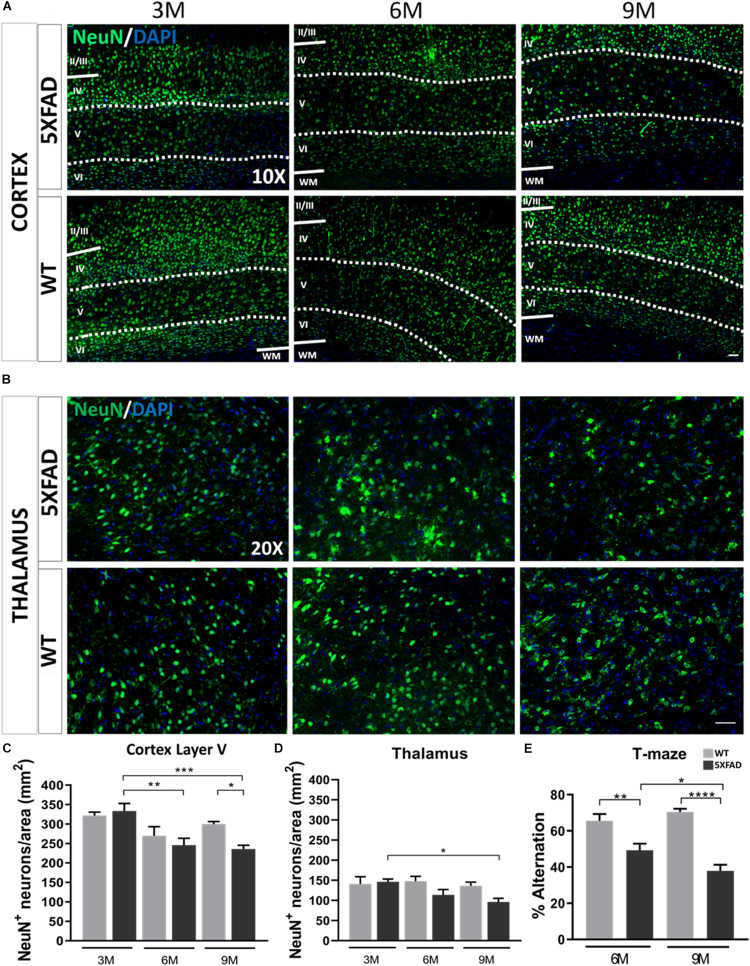
Neuronal loss and cognitive decline in 5XFAD mice. **(A)** Immunofluorescence staining of cortical areas from 5XFAD and WT mice with neuronal marker NeuN^+^ (green) labeling neurons. Cell nuclei are counterstained with DAPI (blue). Cortical layer V is indicated with dotted lines and limits of other cortical layers are indicated with white lines. **(B)** Immunofluorescence staining of thalamic areas from 5XFAD and WT mice with neuronal marker NeuN^+^ (green) labeling neurons. **(C)** Quantification of the mean number of neurons in cortical layer V indicates neuronal loss in 6- and 9-months-old compared to 3-months-old 5XFAD mice. **(D)** In the thalamus, quantification of the mean number of neurons shows there is neuronal loss in 9-months-old 5XFAD compared to 3-months-old 5XFAD. [One-way ANOVA followed by Sidak’s multiple comparisons test, 5XFAD mice/age (*n* = 6), WT mice/age (*n* = 6)]. **(E)** T-maze behavioral test indicates reduced alternation percentage in 5XFAD mice in both age groups [unpaired *t*-test, 6M 5XFAD mice (*n* = 20), 6M WT mice (*n* = 18), 9M 5XFAD mice (*n* = 12), 9M WT mice (*n* = 12)]. Graphs show the mean and error bars indicate the standard error of the mean (SEM). Significance is given as: *p = 0.0332, **p = 0.0021, ***p = 0.0002, ****p < 0.0001. Scale bars = 50 μm in **(A,B)**.

Aβ plaques in the brain can contribute to loss of function and neuronal death either directly or indirectly by disrupting the communication between neurons (Parihar and Brewer, 2010). Therefore, we investigated whether mature neurons were affected by the AD pathology in this mouse model. Since the majority of Aβ plaques were localized in the cortical layer V and thalamus, as shown above, we assessed possible neuronal loss in these areas. Neurons were counted from an area of 260 and 346 mm^2^ in the cortical layer V and thalamus, respectively. Immunostaining with NeuN antibody (Figures 2A–D) showed that 3-months-old 5XFAD and WT mice had similar numbers of neurons in cortical layer V (5XFAD: 333 ± 19 NeuN^+^ cells per area, WT: 322 ± 9 NeuN^+^ cells per area). However, 6- and 9-months-old 5XFAD mice had significantly lower numbers of mature neurons (246 ± 18 NeuN^+^ cells per area and 236 ± 10, respectively) compared to those at 3-months of age (333 ± 19 NeuN^+^ cells per area). Also, the number of mature neurons in 9-months-old 5XFAD mice was significantly lower (236 ± 10 NeuN^+^ cells per area) compared to WT mice of the same age (300 ± 6 NeuN^+^ cells per area). In the thalamus, neuronal loss was significant only in 9-months-old (96 ± 9 NeuN^+^ cells per area) compared to 3-months-old 5XFAD mice (146 ± 7 NeuN^+^ cells per area). Taken together, these data indicate, progressive neuronal loss in cortical layer V and to a lesser degree in the thalamus, associated with progressive Aβ deposition in older 5XFAD mice.

Furthermore, to assess the hippocampus-depended working memory and spatial learning of 5XFAD mice we calculated the spontaneous alternation of mice with a T-maze apparatus (Figure 2E). This behavioral test measures the exploratory behavior of animals, i.e., the preference to enter a new arm of the maze rather than the arm they have visited before. 5XFAD mice at 6 months of age showed reduced alternation compared to their age-matched WT controls. Similarly, 9-months-old 5XFAD mice showed reduced alternation compared to WT controls of the same age, as well as compared to 6-months-old 5XFAD mice. Thus, 6- and 9-months-old 5XFAD mice show progressive deficits in memory function due to hippocampal impairment.

### Increased Gliosis in the Brain of Older 5XFAD Mice

A major contributor to neurodegeneration in AD is also neuroinflammation, which is known to be associated with the progression of the disease. Astrocytes and microglia surround Aβ plaques and become reactive propagating inflammatory signals (Fakhoury, 2017). Therefore, anti-GFAP and anti-Iba1 antibodies were used to evaluate the degree of microglia activation and astrogliosis (Kamphuis et al., 2014; Navarro et al., 2018).

**FIGURE 3:**
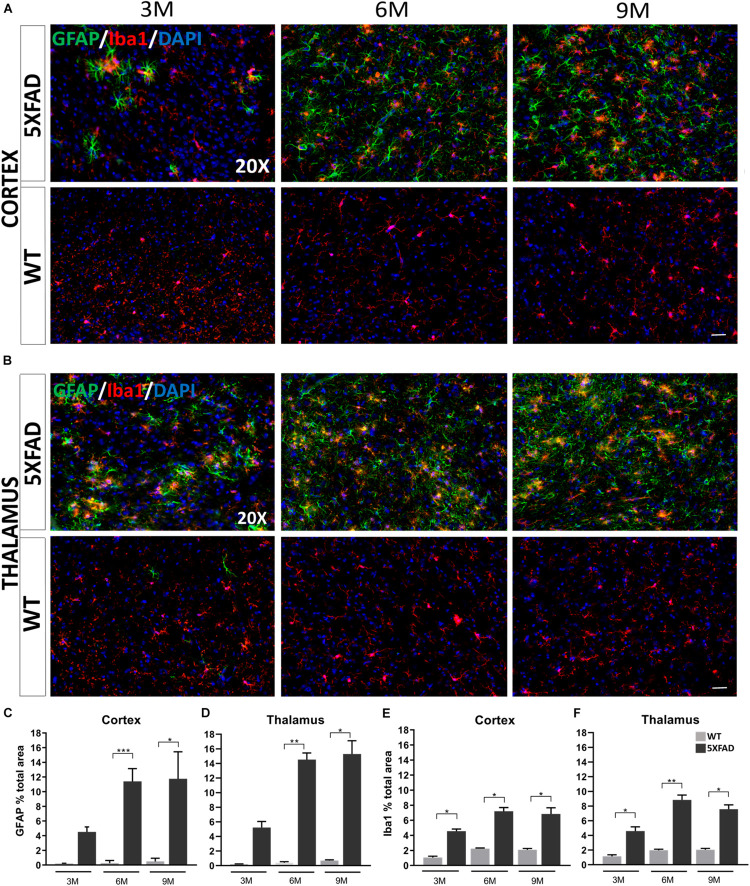
Progressive inflammation and astrogliosis in the RSP, MOp and MOs areas of cortical layer V and in the PO and VPM nucleus of the thalamus in 5XFAD mice. **(A,B)** Double immunofluorescence staining of cortical and thalamic areas from 5XFAD and WT mice, with astrocytic marker, GFAP (green) and microglial marker, Iba1 (red). 5XFAD mice showed increased gliosis in both brain areas in older ages, while WT mice showed no astrogliosis. Quantification of the percentage of the total area covering GFAP^+^ astrocytes **(C,D)** and Iba1^+^ microglia **(E,F)** in both brain areas confirmed increased astrogliosis reaching plateau in 9-months-old 5XFAD mice. The statistical analysis was performed by one-way ANOVA followed by Kruskal-Wallis multiple comparisons test [5XFAD mice/age (*n* = 6), WT mice/age (*n* = 6)]. Graphs show the mean and error bars indicate the standard error of the mean (SEM). Significance is given as: *p = 0.0332, **p = 0.0021, ***p = 0.0002. Scale bars = 50 μm in **(A,B)**.

Immunohistochemistry experiments in 5XFAD mice have revealed some morphological characteristics of glial cells in both brain areas at each age [cortical layer V: retrosplenial (RSP) area, primary and secondary motor areas (MOp, MOs); thalamus: posterior complex (PO) and ventral posteromedial (VPM) nucleus of the thalamus]. Astrocytes in the cortex and thalamus of 3-months-old 5XFAD mice were moderately reactive with an apparent GFAP upregulation (Figures 3A,B). In addition, at the ages of 6 and 9 months, 5XFAD mice showed severe reactive astrogliosis in both brain areas compared to the 3-months-old 5XFAD mice, with GFAP upregulation, cytoskeletal hypertrophy and overlap of astrocytic processes. In contrast, reactive astrocytes were extremely rare in WT mice at all ages. Likewise, microglia at all ages of 5XFAD mice, were shown to be in an activated state, characterized by cell body hypertrophy and shortened processes, in contrast to WT littermates showing lack of microglia activation.

By quantifying the percentage of the total area covered by astrocytes and microglia in the immunostaining images, we confirmed that in both brain areas the levels of GFAP^+^ astrocytes in 6- and 9-months old 5XFAD mice were increased (cortex: 11.39% ± 0.71 and 11.75% ± 1.49, respectively, thalamus: 14.52% ± 0.90 and 15.29% ± 1.82, respectively) compared to 3-months-old 5XFAD mice (cortex: 4.52% ± 0.67, thalamus: 5.23% ± 0.81), whereas the levels were similar when comparing 6- and 9-months-old mice (Figures 3C,D). As expected, WT mice of all ages showed very low levels of GFAP^+^ immunoreactivity in the cortex and thalamus.

Furthermore, in the cortex and thalamus, Iba1^+^ microglia levels (Figures 3E,F) were slightly increased at 6- and 9-months-old 5XFAD mice (cortex: 7.20% ± 0.49 and 6.84% ± 0.81 respectively, thalamus: 8.84% ± 0.65 and 7.58% ± 0.57, respectively) compared to the 3-months-old mice (cortex: 4.55% ± 0.28, thalamus: 4.59% ± 0.57). However, the levels of Iba1^+^ microglia were similar among 6- and 9-months-old 5XFAD mice. In both the cortex and thalamus 5XFAD mice of all age groups showed significantly increased levels of Iba1^+^ microglia (cortex: 3M: 4.55% ± 0.28, 6M: 7.20% ± 0.49, 9M: 6.84% ± 0.81; thalamus: 3M: 4.59% ± 0.57, 6M: 8.84% ± 0.65, 9M: 7.58% ± 0.57) compared to their age-matched WT littermates (cortex: 3M: 1.05% ± 0.17, 6M: 2.24% ± 0.08, 9M: 2.07% ± 0.17; thalamus: 3M: 1.15% ± 0.21, 6M: 1.97% ± 0.14, 9M: 2.04% ± 0.18).

### Increased Immunoreactivity of Cx43 and Cx30 Around Aβ Plaques in 5XFAD Mice

Astrocytes form highly organized networks in the CNS, maintaining communication via GJs or HCs (Xing et al., 2019). Since reactive astrocytes are involved in inflammatory responses, surrounding Aβ plaques in AD (Perez-Nievas and Serrano-Pozo, 2018) we further investigated the expression of the major astrocytic Cxs (Cx43 and Cx30) which compose the GJs or HCs in 5XFAD mice. However, we cannot exclude that some of the Cx43 could be expressed in microglia or non-GFAP astrocytes (Supplementary Figure 1). We examined their expression in the RSP, MOp and MOs areas of cortical layer V and in the PO and VPM nucleus of the thalamus in 3- and 9-months-old 5XFAD mice, where neuronal loss was observed in older mice.

**FIGURE 4:**
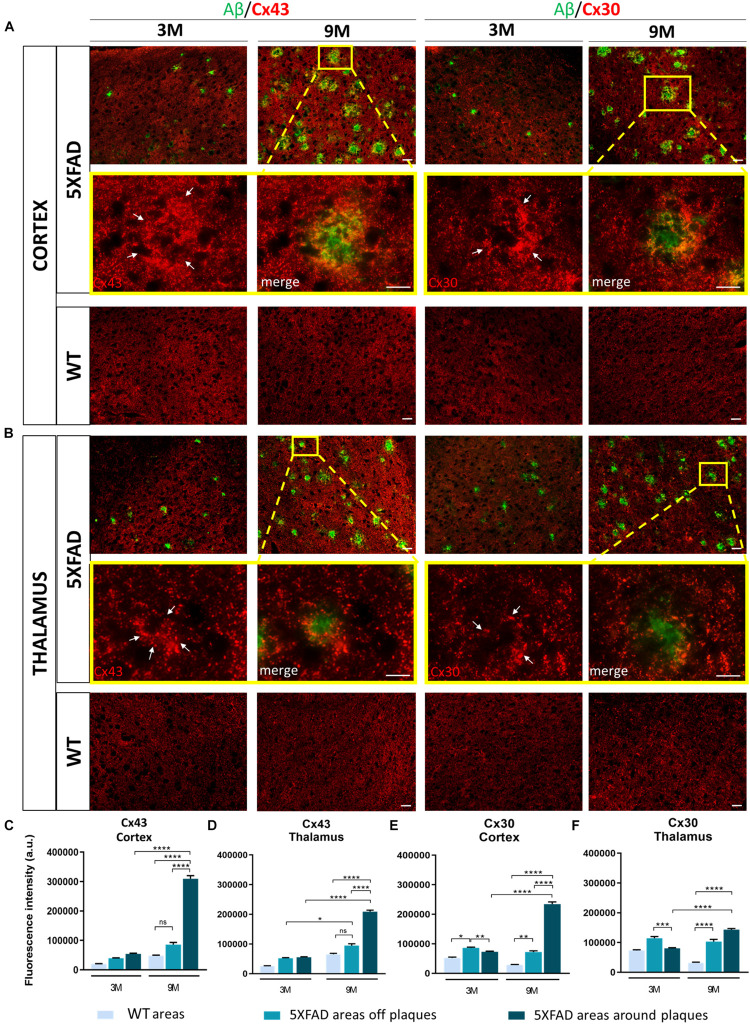
Increased immunoreactivity of Cx43 and Cx30 in areas around Aβ plaques in the RSP, MOp and MOs areas of cortical layer V and in the PO and VPM nucleus of the thalamus of 3 and 9-months-old 5XFAD mice. Double immunofluorescence staining of cortical and thalamic areas from 5XFAD and WT mice, with Aβ antibody (clone 6E10, green) and Cx43 or Cx30 (red). **(A,B)** 5XFAD mice at the age of 9-months showed increased immunoreactivity of Cx43 and Cx30 in the perimeter of Aβ plaques. Higher magnification images clearly show this phenomenon. **(C–F)** Quantification of the fluorescence intensity of Cx43 and Cx30 in areas around and away from Aβ plaques in 5XFAD mice and in areas in WT mice. Increased immunoreactivity of Cx43 and Cx30 was detected in the perimeter of Aβ plaques compared to areas off Aβ plaques in 5XFAD mice and in WT areas. The statistical analysis was performed by one-way ANOVA followed by Kruskal-Wallis multiple comparisons test [5XFAD mice/age (*n* = 6), WT mice/age (*n* = 6)]. Graphs show the mean and error bars indicate the standard error of the mean (SEM). Significance is given as: *p = 0.0332, **p = 0.0021, ***p = 0.0002, ****p < 0.0001. Scale bars = 50 μm in **(A,B)**; 25 μm in higher magnification insets.

Double immunostainings for Aβ/Cx43 and Aβ/Cx30 were performed (Figures 4A,B) and the fluorescence intensity of both Cxs was measured in the area around the perimeter of Aβ plaques and away from Aβ plaques, compared to the corresponding areas of aged-matched WT controls. Results were categorized in three different Cx immunoreactivity profiles around Aβ plaques: increased, unchanged, and decreased immunoreactivity compared to areas away from Aβ plaques. The percentages of Aβ plaques having each of those profiles were calculated (Supplementary Figure 2).

In the cortex and thalamus, Cx43 immunoreactivity was higher in 9-months-old 5XFAD mice and especially around Aβ plaques (cortex: 309,658 ± 10,389 a.u., thalamus: 209,265 ± 4,893 a.u.) compared to 3-months-old mice (cortex: 53,608 ± 2,737 a.u., thalamus: 55,192 ± 1,858 a.u.). There was also significantly higher Cx43 immunoreactivity at 9 months around the area of plaques compared to areas away from plaques (cortex: 85,664 ± 7,541 a.u., thalamus: 94,554 ± 6,280 a.u., Figures 4C,D). In contrast, Cx43 immunoreactivity in the corresponding areas of 9-months-old WT mice (cortex: 47,447 ± 2,596 a.u., thalamus: 65,454 ± 3,203 a.u.) and in areas far from Aβ plaques in 5XFAD mice (cortex: 85,664 ± 7,541 a.u., thalamus: 94,554 ± 6,280 a.u.) showed no significant difference, although there was a tendency to increase in 5XFAD non-plaque areas. These results confirmed that Cx43 channels were increased specifically in the microenvironment of Aβ plaques and not in areas that were far away. The expression of Cx43 in 3-months-old 5XFAD mice around plaques in both brain regions (cortex: 53,608 ± 2,737 a.u., thalamus: 55,192 ± 1,858 a.u.) was similar to the expression of Cx43 in areas away from plaques (cortex: 39,093 ± 1,700 a.u., thalamus: 51,797 ± 2,280 a.u.) and in areas of WT mice of the same age (cortex: 19,893 ± 1,134 a.u., thalamus: 25,228 ± 1,565 a.u.) as well as in areas of 9-months-old WT mice (cortex: 47,447 ± 2,596 a.u., thalamus: 65,454 ± 3,203 a.u.). Thus, increased Cx43 expression around Aβ plaques likely occurs at later stages of AD pathology as a result of progressive astrogliosis and neurodegeneration.

Similar to Cx43, Cx30 immunoreactivity (Figures 4E,F) was increased around Aβ plaques in 9-months-old 5XFAD mice (cortex: 234,427 ± 7,560 a.u., thalamus: 143,549 ± 3,405 a.u.) compared to 3-months-old 5XFAD mice (cortex: 72,913 ± 2,382 a.u., thalamus: 80,219 ± 2,478 a.u.). In the cortex, Cx30 immunoreactivity was also increased around Aβ plaques (234,427 ± 7,560 a.u.) compared to areas away from plaques (72,290 ± 3,769 a.u.) and in corresponding areas of WT controls (28,749 ± 1,152 a.u.). However, in the thalamus of 9-month-old 5XFAD mice there was no significant difference between the immunoreactivity of Cx30 around Aβ plaques (143,549 ± 3,405 a.u.) and areas away from Aβ plaques (103,272 ± 7,420 a.u.). Cx30 immunoreactivity in those two areas was significantly increased compared to areas in WT controls (31,984 ± 1,895 a.u.). These results indicate that Cx30 was increased around Aβ plaques in the cortices of aged 5XFAD mice, but this did not occur in the thalamus, where Cx30 was more diffusely increased both around and in areas away from plaques. At 3 months of age, in both brain regions of 5XFAD mice, Cx30 was slightly increased in areas away from plaques (cortex: 86,273 ± 2,519 a.u., thalamus: 114,374 ± 5,635 a.u.) compared to areas around plaques (cortex: 72,913 ± 2,382 a.u., thalamus: 80,219 ± 2,478 a.u.) and areas in WT controls (cortex: 52,192 ± 2,764 a.u., thalamus: 73,084 ± 2,639 a.u.). These findings suggest that both astrocyte Cxs are increased as a result of AD pathology, but Cx43 is more prominent than Cx30 in reactive astrocytes especially around Aβ plaques.

### Increased Cx43 Protein and Unchanged Cx30 Protein Levels in 5XFAD Mice of All Ages

To further corroborate the results of the immunofluorescence experiments, indicating specific alterations of astrocytic Cxs and in particular of Cx43 more than Cx30 in the microenvironment of Aβ plaques in older 5XFAD mice, we investigated the mRNA and protein levels of these two astrocytic Cxs in the cortex and thalamus of these mice.

**FIGURE 5:**
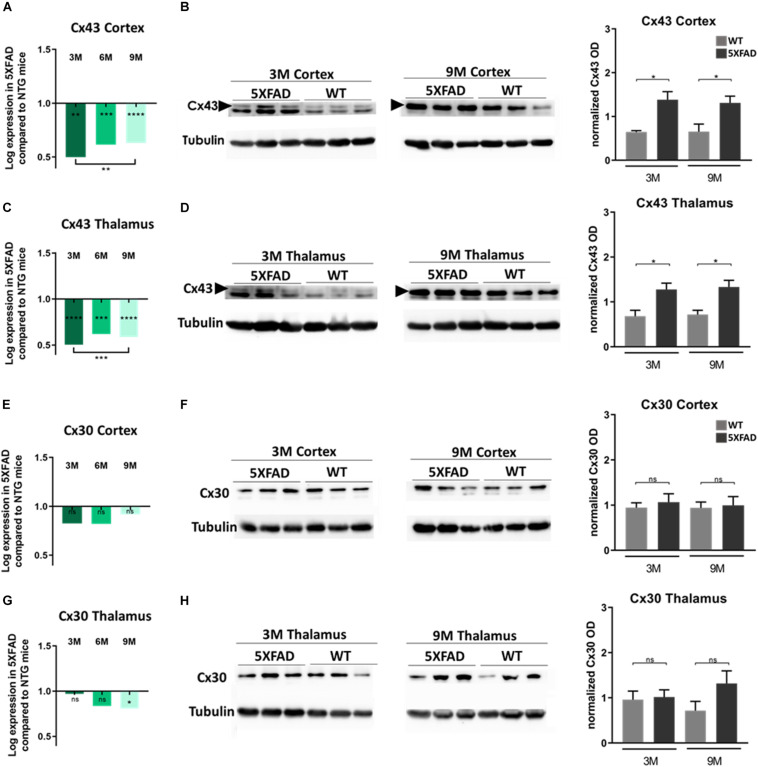
Cx43 and Cx30 mRNA and protein levels in the brain of 5XFAD mice. **(A,C)** Cx43 mRNA levels drop significantly at all ages of 5XFAD mice compared to WT controls in both cortex and thalamus. **(B,D)** However, quantification of immunoblots showed increased protein levels of Cx43 in 3- and 9-months-old 5XFAD mice compared to their WT controls, respectively. **(E,G)** Cx30 mRNA levels were similar at all ages of 5XFAD mice compared to WT controls in the cortex, except in thalamus where a significant drop was shown. **(F,H)** The quantification of immunoblots showed similar levels of Cx30 protein in both brain areas. The asterisks inside the columns of mRNA graphs indicate the *p*-values representing significance of connexin levels in 5XFAD compared to WT controls, while the asterisks outside the columns represent the significance of connexin levels between different age groups of 5XFAD mice. The statistical analysis for mRNA was performed by one-way ANOVA followed by Kruskal-Wallis multiple comparisons test [5XFAD mice/age (*n* = 6), WT mice/age (*n* = 6)], while the immunoblot analysis was performed by unpaired *t*-test [5XFAD mice/age (*n* = 3), WT mice/age (*n* = 3)]. Graphs show the mean and error bars indicate the standard error of the mean (SEM). Significance is given as: *p = 0.0332, **p = 0.0021, ***p = 0.0002, ****p < 0.0001.

Interestingly, in both brain areas, Cx43 mRNA levels in all three age groups of 5XFAD mice, were significantly lower compared to their aged-matched WT controls (Figures 5A,C). Moreover, mRNA levels were significantly lower in 3- compared to 9-months-old 5XFAD mice, in both brain areas but in the thalamus this significance was stronger than in the cortex. In contrast, immunoblot experiments showed increased levels of Cx43 protein in 3- and 9-months-old 5XFAD mice compared to their aged matched WT controls, in the cortex as well as in the thalamus (Figures 5B,D and Supplementary Figure 3, shows original gels), in keeping with the immunostaining results. This inverse correlation between mRNA and protein levels could reflect the complex regulatory mechanisms that underlie transcription and translation such as transcriptional modifications (Yan et al., 2018).

On the other hand, Cx30 mRNA levels in both brain areas from all three age groups of 5XFAD mice showed no significant difference compared to their WT controls, except in the thalamus, where 9-months-old 5XFAD mice showed significantly lower levels of Cx30 mRNA compared to the WT mice (Figures 5E,G). Furthermore, immunoblot analysis of Cx30 protein showed similar levels between all ages of 5XFAD mice compared to the WT controls (Figures 5F,H and Supplementary Figure 3; shows original gels), in line with the mRNA levels.

Generally, mRNA and immunoblot experiments were performed in lysed cortices and thalami, where the average levels of Cx43 and Cx30 were measured in the whole tissue and not specifically around the perimeter of Aβ plaques, which were specifically measured in the immunostainings. Immunoblot results confirmed that Cx43 is the astrocytic Cx that is mostly altered in the context of 5XFAD brain pathology compared to WT mice, as observed by the immunofluorescence experiments as well. Whether Cx43 localization around Aβ plaques is only a consequence of astrogliosis or whether it also contributes to the disease propagation remains to be shown.

### Decreased Immunoreactivity of Cx47 in 5XFAD Mice

Since we found an increased immunoreactivity of Cx43 around Aβ plaques in 9-months-old 5XFAD mice, we further asked whether the major partner of Cx43 at A/O heterotypic GJs, Cx47 (Orthmann-Murphy et al., 2007), would be affected in the course of AD pathology.

**FIGURE 6:**
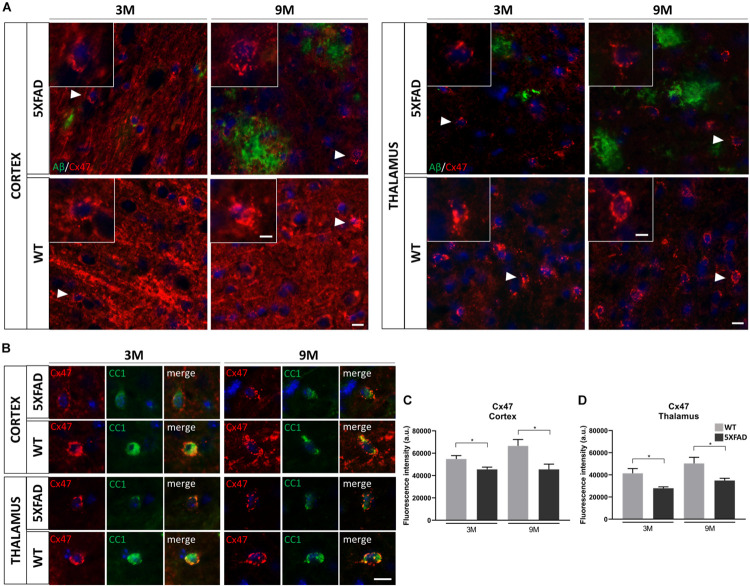
Decreased immunoreactivity of Cx47 in RSP, Mop, and MOs areas of cortical layer V and in the PO and VPM nucleus of the thalamus in 3- and 9-months-old 5XFAD mice. **(A)** Double immunostaining for Aβ/Cx47 in cortical and thalamic areas showed decreased Cx47 immunoreactivity of 5XFAD mice in both ages compared to WT mice. Zoomed images show the Cx47 puncta in more detail. **(B)** Double immunostaining for CC1/Cx47 in cortical and thalamic areas in the presence of Aβ plaques, in 5XFAD and WT mice. Cx47-positive GJ plaques were expressed by mature oligodendrocytes. Cell nuclei were counterstained with DAPI (blue). **(C,D)** Quantification of Cx47 fluorescence intensity confirmed a decreased immunoreactivity as observed in the immunostainings. The statistical analysis was performed by unpaired *t*-test [5XFAD mice/age (*n* = 6), WT mice/age (*n* = 6)]. Graphs show the mean and error bars indicate the standard error of the mean (SEM). Significance is given as: *p = 0.0332. Scale bars = 10 μm in **(A,B)**; 5 μm in higher magnification insets.

Double immunostainings were performed for Cx47 and either Aβ or CC1 (oligodendrocyte marker) in 3- and 9-months-old 5XFAD and control mice. We captured Cx47 immunoreactive oligodendrocytes in the presence of Aβ in the RSP, MOp and MOs areas of cortical layer V and in the PO and VPM nucleus of the thalamus, with some being closer to Aβ plaque microenvironment and some far away. All Cx47-positive puncta were used to measure the fluorescence intensity of Cx47 (Figure 6A). Cx47-positive GJ plaques were mostly localized around CC1-positive cell bodies and processes of mature oligodendrocytes (Figure 6B). These two immunostaining experiments (CC1/Cx47, Aβ/Cx47) showed a decreased Cx47 fluorescence intensity in oligodendrocytes of 3- and 9-months-old 5XFAD mice compared to their WT age-matched controls. Quantification of Cx47 fluorescence intensity (Figures 6C,D) also confirmed that 5XFAD mice at both age groups showed decreased Cx47 immunoreactivity compared to their age-matched controls in the cortex and thalamus (cortex: 3M 5XFAD mice: 45,565 ± 2,032 a.u., 3M WT mice: 54,811 ± 3,066 a.u.,9M 5XFAD mice: 45,600 ± 4,727 a.u., 9M WT mice: 66,579 ± 5,692 a.u.; thalamus: 3M 5XFAD mice: 27,974 ± 1,235 a.u., 3M WT mice: 41,482 ± 4,191 a.u., 9M 5XFAD mice: 34,966 ± 1,975 a.u., 9M WT mice: 50,363 ± 5,543 a.u.). Thus, in contrast to the astrocyte partner Cx43, oligodendrocytic Cx47 shows reduced expression around Aβ plaques, indicating loss of A/O connectivity in the context of AD pathology. Double immunostaining for Cx47 and Cx43 confirmed this finding by showing reduced Cx47 GJ plaques with less colocalization with Cx43 plaques in 9-months-old 5XFAD mice compared to WT mice (Supplementary Figure 4). Interestingly, Cx47 and Cx32 mRNA levels showed no significant difference between 5XFAD and WT mice in both the cortex and thalamus at all ages (Supplementary Figure 5). Also, double immunostaining for Aβ and Cx32 showed a diffuse immunoreactivity of Cx32 which was reduced in 5XFAD mice as well as disruption of Cx32 within and outside Aβ plaques (Supplementary Figure 6).

### Decreased Numbers of Oligodendrocyte Precursors and Mature Oligodendrocytes and Myelin Defects in 5XFAD Mice

Furthermore, we analyzed cells of the oligodendrocyte lineage (excluding some NG2^+^ cells) in the cortex and thalamus of 3- and 9-months-old 5XFAD mice and their age-matched WT littermates, to investigate the reaction of these cells in the presence of Aβ.

**FIGURE 7:**
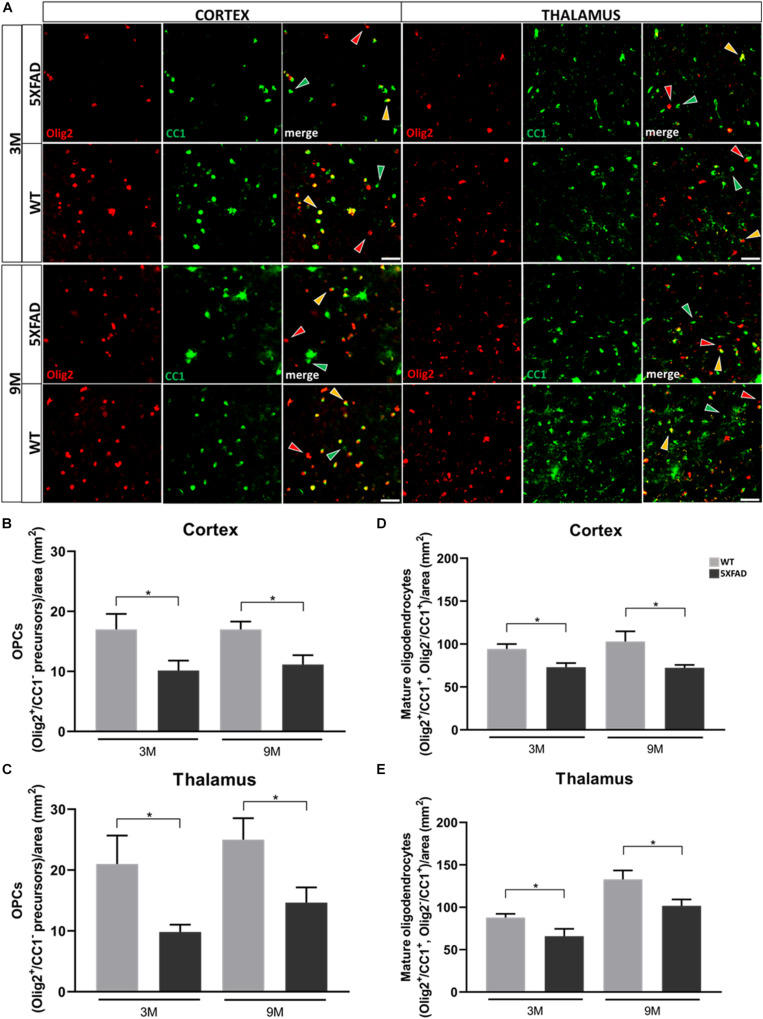
Depletion of OPC and mature oligodendrocyte numbers in 3- and 9-months-old in 5XFAD mice. **(A)** Double immunostaining for Olig2/CC1 in cortical and thalamic areas of 5XFAD and WT mice. Yellow, green and red arrows depict Olig2^+^/CC1^+^ mature oligodendrocytes, Olig2^–^/CC1^+^ mature oligodendrocytes and Olig2^+^/CC1^–^ OPCs, respectively. **(B)** Mean numbers of OPCs in the cortex and thalamus **(C)**. **(D)** Total mean numbers of mature oligodendrocytes (Olig2^+^/CC1^+^, Olig2^–^/CC1^+^ cells) in the cortex and thalamus **(E)**. The statistical analysis was performed by unpaired *t*-test [5XFAD mice/age (*n* = 6), WT mice/age (*n* = 6)]. Graphs show the mean and error bars indicate the standard error of the mean (SEM). Significance is given as: *p = 0.0332. Scale bar = 50 μm.

Double immunostaining was performed for Olig2, a marker of both oligodendrocyte precursors (OPCs) and mature oligodendrocytes, and CC1, a marker of mature oligodendrocytes. Three different populations of oligodendrocytes were observed; Olig2^+^/CC1^+^ and Olig2^–^/CC1^+^ cells, which indicate mature oligodendrocytes, and Olig2^+^/CC1^–^ cells, which indicate OPCs (Figure 7A). Cells were counted from an area of 346 mm^2^. Olig2^+^ cells were not found to be in clusters around Aβ plaques (Supplementary Figure 7). The mean numbers of OPC and mature oligodendrocyte populations were decreased at both ages of 5XFAD compared to control mice, in the cortex and thalamus (Figures 7B–E). These results reveal the loss of mature oligodendrocytes, together with the loss of Cx47 in 5XFAD mice. Therefore, the loss of A/O connectivity could affect the survival of mature oligodendrocytes. Moreover, the loss of OPCs clearly indicates that these cells were impaired due to AD pathology and possibly could not generate adequate numbers of mature oligodendrocytes at least at the level of WT mice.

**FIGURE 8:**
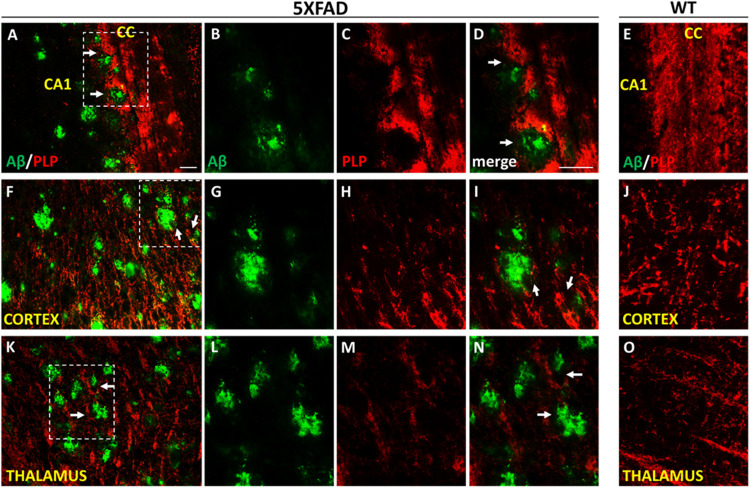
Myelin disruption in the microenvironment of Aβ plaques in 9-months-old 5XFAD mice. Double immunofluorescence staining of corpus callosum (CC), cortex and thalamus of 5XFAD and WT mice with Aβ and myelin marker PLP (myelin proteolipid protein). The myelin structure is disrupted in the area surrounding Aβ plaques (white arrows) in the CC **(A–D)**, cortex **(F–I)** and thalamus **(K–N)** in 5XFAD mice compared to myelin immunoreactivity in the respective areas in WT mice **(E,J,O)** which appears normal. Magnification x20 in **(A,F,K)**. Magnification x40 in **(B–E,G–J,L–O)**. Scale bars = 100 μm.

We further looked myelination at the microenvironment of Aβ plaques by performing Aβ/PLP double immunostaining in 9-months-old 5XFAD and WT mice. Myelin proteolipid protein (PLP) is a major component of the CNS myelin (Yool et al., 2000). We observed myelin defects in areas surrounding Aβ plaques in the cortex, corpus callosum and thalamus (Figure 8), which appears to be a consequence of OPCs and mature oligodendrocytes loss.

## Discussion

In this study we suggest the implication of glial Cxs in the progression of AD. The increased Cx43 immunoreactivity at the microenvironment of Aβ plaques, reflects astrogliosis, while the reduced Cx47 immunoreactivity indicates loss of connection between astrocytes and oligodendrocytes. Moreover, we suggest that the disruption of O/A GJs could be a cause for the depletion of OPCs and mature oligodendrocytes in the 5XFAD mouse model of AD. The disruption of the pan-glial syncytium together with astrogliosis could favor A/A connectivity and/or the shift of Cx43 GJs into HCs, with deleterious consequences in neuronal homeostasis.

The 5XFAD transgenic mouse model is an excellent model for the investigation of familial AD. It combines three mutations in the APP and two mutations in the PSEN1 gene to ensure rapid development of Aβ plaques and especially an increase of Aβ_42_ in the cerebrum (Oakley et al., 2006). Therefore, this model provides an advantage for studying AD pathology over other AD mouse models which develop Aβ deposition at a slower rate (Games et al., 1995; Hsiao et al., 1996; Sturchler-Pierrat et al., 1997; Jankowsky et al., 2004; Radde et al., 2006). However, this model is characterized by the absence of neurofibrillary tangles, thus the disease pathology is partially represented, which can be a drawback in understanding the disease mechanisms. The development of both Aβ and tau pathology in AD models depicts their interplay during the development and progression of the disease, thus demonstrating the AD phenotypic alterations in a more comprehensive way (Lewis et al., 2001; Oddo et al., 2003; Boutajangout et al., 2004; Bolmont et al., 2007).

Extracellular Aβ plaques appear at around 2 months of age in 5XFAD mice (Oakley et al., 2006). Plaques are initially found in the cortex and then spread into other brain areas, as deposition rapidly increases with age (Maarouf et al., 2013), which we confirmed in this study. Neuronal loss has also been observed at 6 months of age in cortical layer V (Oakley et al., 2006; Eimer and Vassar, 2013). We confirmed loss of mature neurons starting at 6 and continuing at 9 months of age in cortical layer V, an area with the heaviest deposition and largest size of Aβ plaques compared to other cortical regions. We showed that 5XFAD mice exhibited cognitive deficits compared to WT mice at the age of 6 and 9 months as shown by the T-maze behavioral test, possibly indicating that neuronal loss at those ages causes memory deficits. Spatial memory impairment was also observed by others at 4, 5, and 6 months of age (Oakley et al., 2006; Ohno et al., 2006; Devi and Ohno, 2010; Jawhar et al., 2012; Xiao et al., 2015). Furthermore, in 5XFAD mice, gliosis also appears early in the brain and progresses with age due to the early and aggressive amyloid deposition, as we observed in our experiments. We observed proliferation of reactive GFAP^+^ astrocytes and activated Iba1^+^ microglia from 3 to 9 months of age, which is in parallel with the amyloid load profile in each age. Reactive astrocytes and microglia surround Aβ plaques inducing inflammation, in human and rodent brains with the AD pathology (Nagy et al., 1996; Sturchler-Pierrat et al., 1997; Oakley et al., 2006).

We demonstrate here that astrocytic Cxs (Cx43 and Cx30) and especially Cx43, show increased immunoreactivity around the perimeter of Aβ plaques in the cortex and thalamus of older 5XFAD mice. This resonates to the fact that in older mice, Aβ plaques are larger, the inflammatory response is higher and generally the pathology is more severe. This finding is in concordance with other studies, previously performed in post-mortem human samples and mouse models of AD (Nagy et al., 1996; Mei et al., 2010). Moreover, our immunoblot analysis demonstrated that the total Cx43 protein levels increased at both ages in 5XFAD mice compared to their controls. The increased protein levels of Cx43 together with the immunostaining results clearly confirmed the increased Cx43 expression at the microenvironment of Aβ plaques in older ages, in association with disease progression. Likewise, Cx30 was also increased within the Aβ plaque microenvironment in older mice but immunoblot results showed no difference in Cx30 protein levels at both ages compared to their controls. This may be related to the fact that Cx30 is the dominant Cx isoform and more diffusely expressed in gray matter astrocytes (Griemsmann et al., 2015). These results indicate that Cx43 is more strongly associated with progression of AD than Cx30. Furthermore, when Cx43 was analyzed at the transcriptional level, it was surprisingly downregulated at all ages in 5XFAD mice, while protein levels were increased as mentioned previously. This might be due to transcriptional modifications that hinder the transcription process. The downregulation of Cx43 gene expression might be due to inflammatory mediators. It was reported that the activation of the JNK pathway through TNF-α, reduced Cx43 gene promoter activation (Yan et al., 2018). Also, the presence of microglia and their interaction with astrocytes in an inflammatory environment, like AD, could contribute to the downregulation of Cx43 gene. Reduction of astrocytic Cx43 mRNA levels occurred after treating astrocyte/microglia co-cultures but not astrocyte-rich cultures, with interferon (IFN)γ or IL-17, implying that microglia were responsible for this reduction. The authors depicted an IFNγ-mediated microglial release of IL-1β as the causal mechanism (Watanabe et al., 2016). Furthermore, JNK and NF-κB pathways were activated via IL-1β in astrocytes (Wu et al., 2004), therefore the reduction in Cx43 mRNA could be caused through those pathways.

Moreover, due to the activation of astrocytes in a neuroinflammatory environment, Cx43 might shift its expression from GJs to HCs (Retamal et al., 2007; Kielian, 2008). The combination of TNF-a and IL-1β in astrocyte cultures of mice, caused the reduction of GJs and the upregulation of Cx43 HC activity (Chen et al., 2014; Xu et al., 2019). Prolonged activation of Cx43 HCs in the presence of Aβ plaques, was reported in astrocytes of APP/PS1 mice and especially those near plaques (Yi et al., 2016). Our results could indicate a pathological pathway in the 5XFAD mouse model of AD, involving the opening of Cx43 HCs, but this requires further investigation. Activated microglia can release pro-inflammatory cytokines, which increase astrocytic Cx43 HC opening (Orellana et al., 2009). Increase in [Ca^2+^]_i_ could trigger the openings of Cx43 HCs, allowing Ca^2+^ entrance, thus establishing the maintenance of upregulated [Ca^2+^]_i_ in astrocytes. Then, release of gliotransmitters occurs via HCs leading to increased extracellular glutamate and ATP (Orellana et al., 2011b). In the presence of Aβ, glutamate binds to neuronal NMDA receptors and ATP to purinergic (P2X) receptors, which generate the enhancement of intraneuronal Ca^+2^, causing neuronal dysfunction and synaptic loss (Talantova et al., 2013), neuritic alterations (Kuchibhotla et al., 2008), and oxidative stress (Lin and Beal, 2006). ATP also stimulates the activation of P2×7 receptors in astrocytes and microglia triggering the release of IL-1β which is toxic to neurons (Pelegrin and Surprenant, 2009). Finally, glutamate and ATP trigger the opening of neuronal Panx1 HCs (Pelegrin and Surprenant, 2006), which allow further Ca^2+^ entry causing neuronal death and activation of neurotoxic cascades (Orellana et al., 2011a).

Furthermore, we showed that Cx47 immunoreactivity was reduced in mature oligodendrocytes in the presence of Aβ, while Cx43 immunoreactivity was increased around Aβ plaques. We also confirmed this finding by showing reduced colocalization of Cx47 with Cx43 GJ plaques (Supplementary Figure 4). Such findings were also observed in chronic MS lesions in both white and gray matter. In particular, Cx43 GJ plaques showed increased density but Cx47 GJ plaques were decreased in numbers, while there was a decreased colocalization of Cx47 and Cx43 in the MS normal appearing white matter and in periplaque areas (Markoullis et al., 2012b, 2014). Loss of Cx47 is observed not only in MS (Masaki et al., 2013) but also in other neuroinflammatory disorders including Neuromyelitis optica and Balo’s disease, an MS variant (Masaki et al., 2012) as a result of astrogliosis, which compromises Cx47.

These findings indicate a possible mechanism for the spreading of the demyelinating disease, which is the disruption of the O/A GJs caused by impairment of the pan-glial GJ network disturbing primarily the oligodendrocytes, which possibly occurs in AD as well. These contrasting patterns of Cx47 and Cx43 were also observed in a chronic EAE mouse model of MS, showing that astrocytes are the major determinants for spreading GJ pathology (Markoullis et al., 2012a). Upregulation of Cx43 was also observed in other chronic stage EAE models (Roscoe et al., 2007a,b), in a mouse model of Parnkinson’s disease (Rufer et al., 1996) and in epilepsy in humans (Aronica et al., 2001; Fonseca et al., 2002). Also, increased Cx43 and reduced Cx47 immunoreactivity were observed at the progressive and end stages of an amyotrophic lateral sclerosis mouse model (Cui et al., 2014). We suggest that in the 5XFAD mouse model of AD, the increase of Cx43 but not Cx47 immunoreactivity could possibly indicate the presence of Cx43 in A/A and not in O/A channels, leading to demyelination, just like in chronic EAE and MS (Markoullis et al., 2012a).

The loss of A/O connectivity could also affect the survival of mature oligodendrocytes in 5XFAD mice, which were found to be depleted in our study. Loss of O/A GJs formed by Cx47 and Cx43, may reflect the dependency of Cx47 expression on the presence of Cx43 on the cell membrane (May et al., 2013). The loss of O/A GJs is predicted to negatively affect the survival and function of oligodendrocytes. The significance of O/A connections is highlighted by the fact that Cx43 mutations which dissociate A/O GJs, also induce demyelination (Paznekas et al., 2003). Severe demyelination is also caused in Cx43/Cx30 double KO mice which depict disruption of O/A GJs (Lutz et al., 2009). AD models show different alterations in myelination patterns and oligodendrocyte capacity, prior to the appearance of Aβ plaques depending on the different mutations they carry (Desai et al., 2009, 2010; Cai and Xiao, 2016). OPCs and oligodendrocytes are the most vulnerable cells of the CNS due to the complexity of their differentiation mechanism and their metabolic program (Papaneophytou et al., 2019). Indeed, the loss of OPCs in 5XFAD mice indicates that these cells become vulnerable in the disease course and possibly cannot generate new populations of myelinating oligodendrocytes to compensate for the depletion of mature oligodendrocytes observed in this model. It is possible that the repair of damaged OPCs could not be achieved, thus mature oligodendrocytes could not be generated, causing myelin breakdown (Roher et al., 2002; Sjöbeck et al., 2005). Increased differentiation of OPCs into mature oligodendrocytes was reported in the early stages of AD, as a compensatory mechanism for myelin loss (Behrendt et al., 2013). However, we did not observe this in 3-months-old 5XFAD mice. This could possibly occur in an earlier stage, but this requires further investigation. Also, the demyelination and remyelination processes have not been studied extensively in the 5XFAD model and thus require further thorough examination. Myelin basic protein (MBP) was found to be significantly reduced in 1-month-old 5XFAD compared to WT mice, indicating myelin loss as an early event (Wu et al., 2018). Also, 12-months-old 5XFAD mice exhibited disruption of myelin focally around Aβ plaques on the edge of corpus callosum (Kaya et al., 2020). We also observed myelin defects at the microenvironment of Aβ plaques in 9-months-old 5XFAD mice, which explains the decrease in the numbers of oligodendrocytes. Direct Aβ toxic effects (Lee et al., 2004; Desai et al., 2010; Agosta et al., 2014) and Aβ oligomer toxicity are mechanisms that influence proliferation, survival, and function of OPCs and oligodendrocytes, causing white matter degeneration and impaired myelin maintenance and remyelination (Horiuchi et al., 2012). Moreover, inflammatory signals exacerbate Aβ-induced demyelination and oligodendrocyte damage (Jantaratnotai et al., 2003).

## Conclusion

Our study reports the increased Cx43 immunoreactivity in the immediate vicinity of Aβ plaques, for the first time in the 5XFAD mouse model of AD. It also provides evidence of decreased Cx47 and Cx32 immunoreactivity suggesting a reduction in Cx43-Cx47 GJs, indicating the loss of A/O coupling. The loss of interaction between astrocytes and oligodendrocytes as well as Aβ pathology could be factors that induce loss of OPCs and mature oligodendrocytes with myelin defects as well in this model. The disruption of glial syncytium and astrogliosis favors A/A coupling and possibly the activation of Cx43 HCs, with detrimental effects in neuronal survival. All these provide evidence that glial Cxs might contribute to the progression of AD. These results warrant further study of oligodendrocyte Cxs in AD pathophysiology and progression.

## Data Availability Statement

All datasets presented in this study are included in the article/Supplementary Material.

## Ethics Statement

The animal study was reviewed and approved by the Cyprus Government’s Veterinary Services 1417 Nicosia, Cyprus.

## Author Contributions

SA designed and performed the experiments, analyzed and interpreted data, and wrote the manuscript. IK performed experiments, supervised the study, and reviewed the manuscript. MS assisted with data analysis and interpretation. IS and EG provided protocols and guided with experimental procedures. SP supervised the study and reviewed the manuscript. KK contributed to conception, study design and had critical input in the preparation and review of the manuscript. All authors contributed to the article and approved the submitted version.

## Conflict of Interest

The authors declare that the research was conducted in the absence of any commercial or financial relationships that could be construed as a potential conflict of interest.

## References

[B1] Agosta F. Libera D. D. Spinelli E. G. Finardi A. Canu E. Bergami A. (2014). Myeloid microvesicles in cerebrospinal fluid are associated with myelin damage and neuronal loss in mild cognitive impairment and alzheimer disease. *Ann. Neurol.* 76 813–825. 10.1002/ana.24235 25087695

[B2] Aronica E. Gorter J. A. Jansen G. H. Leenstra S. Yankaya B. Troost D. (2001). Expression of connexin 43 and connexin 32 gap-junction proteins in epilepsy-associated brain tumors and in the perilesional epileptic cortex. *Acta Neuropathol.* 101 449–459. 10.1007/s004010000305 11484816

[B3] Balez R. Ooi L. (2016). Getting to NO Alzheimer’s disease: neuroprotection versus neurotoxicity mediated by nitric oxide. *Oxid. Med. Cell. Longev.* 2016:3806157. 10.1155/2016/3806157 26697132 PMC4677236

[B4] Bautista W. McCrea D. A. Nagy J. I. (2014). Connexin36 identified at morphologically mixed chemical/electrical synapses on trigeminal motoneurons and at primary afferent terminals on spinal cord neurons in adult mouse and rat. *Neuroscience* 263 159–180. 10.1016/j.neuroscience.2013.12.057 24406437 PMC3951135

[B5] Behrendt G. Baer K. Buffo A. Curtis M. A. Faull R. L. Rees M. I. (2013). Dynamic changes in myelin aberrations and oligodendrocyte generation in chronic amyloidosis in mice and men. *Glia* 61 273–286. 10.1002/glia.22432 23090919

[B6] Belousov A. B. Nishimune H. Denisova J. V. Fontes J. D. (2018). A potential role for neuronal connexin 36 in the pathogenesis of amyotrophic lateral sclerosis. *Neurosci. Lett.* 666 1–4. 10.1016/j.neulet.2017.12.027 29246791 PMC5805564

[B7] Bolmont T. Clavaguera F. Meyer-Luehmann M. Herzig M. C. Radde R. Staufenbiel M. (2007). Induction of tau pathology by intracerebral infusion of amyloid-β-containing brain extract and by amyloid-β deposition in APP x tau transgenic mice. *Am. J. Pathol.* 171 2012–2020. 10.2353/ajpath.2007.070403 18055549 PMC2111123

[B8] Boutajangout A. Authelet M. Blanchard V. Touchet N. Tremp G. Pradier L. (2004). Characterisation of cytoskeletal abnormalities in mice transgenic for wild-type human tau and familial Alzheimer’s disease mutants of APP and presenilin-1. *Neurobiol. Dis.* 15 47–60. 10.1016/j.nbd.2003.09.007 14751770

[B9] Brand-Schieber E. Werner P. Iacobas D. A. Iacobas S. Beelitz M. Lowery S. L. (2005). Connexin43, the major gap junction protein of astrocytes, is down-regulated in inflamed white matter in an animal model of multiple sclerosis. *J. Neurosci. Res.* 80 798–808. 10.1002/jnr.20474 15898103 PMC1226319

[B10] Cai Z. Xiao M. (2016). Oligodendrocytes and Alzheimer’s disease. *Int. J. Neurosci.* 126 97–104. 10.3109/00207454.2015.1025778 26000818

[B11] Chen G. Park C. K. Xie R. G. Berta T. Nedergaard M. Ji R. R. (2014). Connexin-43 induces chemokine release from spinal cord astrocytes to maintain late-phase neuropathic pain in mice. *Brain* 137 2193–2209. 10.1093/brain/awu140 24919967 PMC4107738

[B12] Cragnolini A. Lampitella G. Virtuoso A. Viscovo I. Panetsos F. Papa M. (2020). Regional brain susceptibility to neurodegeneration: What is the role of glial cells? *Neural Regen. Res.* 15 838–842. 10.4103/1673-5374.268897 31719244 PMC6990768

[B13] Cruz N. F. Ball K. K. Dienel G. A. (2010). Astrocytic gap junctional communication is reduced in amyloid-β-treated cultured astrocytes, but not in Alzheimer’s disease transgenic mice. *ASN Neuro* 2:e00041. 10.1042/AN20100017 20730033 PMC2922840

[B14] Cui Y. Masaki K. Yamasaki R. Imamura S. Suzuki S. O. Hayashi S. (2014). Extensive dysregulations of oligodendrocytic and astrocytic connexins are associated with disease progression in an amyotrophic lateral sclerosis mouse model. *J. Neuroinflammation* 11:42. 10.1186/1742-2094-11-42 24597481 PMC4016493

[B15] Deacon R. M. J. Rawlins J. N. P. (2006). T-maze alternation in the rodent. *Nat. Protoc.* 1 7–12. 10.1038/nprot.2006.2 17406205

[B16] Delekate A. Füchtemeier M. Schumacher T. Ulbrich C. Foddis M. Petzold G. C. (2014). Metabotropic P2Y1 receptor signalling mediates astrocytic hyperactivity *in vivo* in an Alzheimer’s disease mouse model. *Nat. Commun.* 5:5422. 10.1038/ncomms6422 25406732

[B17] Desai M. K. Mastrangelo M. A. Ryan D. A. Sudol K. L. Narrow W. C. Bowers W. J. (2010). Early oligodendrocyte/myelin pathology in Alzheimer’s disease mice constitutes a novel therapeutic target. *Am. J. Pathol.* 177 1422–1435. 10.2353/ajpath.2010.100087 20696774 PMC2928974

[B18] Desai M. K. Sudol K. L. Janelsins M. C. Mastrangelo M. A. Frazer M. E. Bowers W. J. (2009). Triple-transgenic Alzheimer’s disease mice exhibit region-specific abnormalities in brain myelination patterns prior to appearance of amyloid and tau pathology. *Glia* 177 1422–1435. 10.1002/glia.20734 18661556 PMC2584762

[B19] Devi L. Ohno M. (2010). Phospho-eIF2α level is important for determining abilities of BACE1 reduction to rescue cholinergic neurodegeneration and memory defects in 5XFAD mice. *PLoS One* 5:e12974. 10.1371/journal.pone.0012974 20886088 PMC2944882

[B20] Eimer W. A. Vassar R. (2013). Neuron loss in the 5XFAD mouse model of Alzheimer’s disease correlates with intraneuronal Aβ42 accumulation and Caspase-3 activation. *Mol. Neurodegener.* 8:2. 10.1186/1750-1326-8-2 23316765 PMC3552866

[B21] Eugenin E. A. Basilio D. Sáez J. C. Orellana J. A. Raine C. S. Bukauskas F. (2012). The role of gap junction channels during physiologic and pathologic conditions of the human central nervous system. *J. Neuroimmune Pharmacol.* 7 499–518. 10.1007/s11481-012-9352-5 22438035 PMC3638201

[B22] Fakhoury M. (2017). Microglia and astrocytes in Alzheimer’s disease: implications for therapy. *Curr. Neuropharmacol.* 16 508–518. 10.2174/1570159x15666170720095240 28730967 PMC5997862

[B23] Fonseca C. G. Green C. R. Nicholson L. F. B. (2002). Upregulation in astrocytic connexin 43 gap junction levels may exacerbate generalized seizures in mesial temporal lobe epilepsy. *Brain Res.* 929 105–116. 10.1016/S0006-8993(01)03289-9 11852037

[B24] Games D. Adams D. Alessandrini R. Barbour R. Borthelette P. Blackwell C. (1995). Alzheimer-type neuropathology in transgenic mice overexpressing V717F β-amyloid precursor protein. *Nature* 523–527. 10.1038/373523a0 7845465

[B25] Giaume C. Koulakoff A. Roux L. Holcman D. Rouach N. (2010). Astroglial networks: a step further in neuroglial and gliovascular interactions. *Nat. Rev. Neurosci.* 11 87–99. 10.1038/nrn2757 20087359

[B26] Griemsmann S. Höft S. P. Bedner P. Zhang J. Von Staden E. Beinhauer A. (2015). Characterization of panglial gap junction networks in the thalamus, neocortex, and hippocampus reveals a unique population of glial cells. *Cereb. Cortex* 25 3420–3433. 10.1093/cercor/bhu157 25037920 PMC4585496

[B27] Griñán-Ferré C. Sarroca S. Ivanova A. Puigoriol-Illamola D. Aguado F. Camins A. (2016). Epigenetic mechanisms underlying cognitive impairment and Alzheimer disease hallmarks in 5XFAD mice. *Aging* 8 664–684. 10.18632/aging.100906 27013617 PMC4925821

[B28] Haass C. Selkoe D. J. (2007). Soluble protein oligomers in neurodegeneration: lessons from the Alzheimer’s amyloid β-peptide. *Nat. Rev. Mol. Cell Biol.* 8 101–112. 10.1038/nrm2101 17245412

[B29] Hardy J. Selkoe D. J. (2002). The Amyloid Hypothesis of Alzheimer’s Disease: progress and Problems on the Road to Therapeutics. *Science* 297 353–357. 10.1126/science.1072994 12130773

[B30] Haughey N. J. Mattson M. P. (2003). Alzheimer’s amyloid β-peptide enhances ATP/Gap junction-mediated calcium-wave propagation in astrocytes. *Neuromol. Med.* 3 173–180. 10.1385/nmm:3:3:173 12835512

[B31] Horiuchi M. Maezawa I. Itoh A. Wakayama K. Jin L. W. Itoh T. (2012). Amyloid β1-42 oligomer inhibits myelin sheet formation in vitro. *Neurobiol. Aging* 33 499–509. 10.1016/j.neurobiolaging.2010.05.007 20594620 PMC3013291

[B32] Hsiao K. Chapman P. Nilsen S. Eckman C. Harigaya Y. Younkin S. (1996). Correlative memory deficits, Aβ elevation, and amyloid plaques in transgenic mice. *Science* 50 793–800. 10.1126/science.274.5284.99 8810256

[B33] Jankowsky J. L. Fadale D. J. Anderson J. Xu G. M. Gonzales V. Jenkins N. A. (2004). Mutant presenilins specifically elevate the levels of the 42 residue β-amyloid peptide *in vivo*: evidence for augmentation of a 42-specific γ secretase. *Hum. Mol. Genet.* 13 159–170. 10.1093/hmg/ddh019 14645205

[B34] Jantaratnotai N. Ryu J. K. Kim S. U. McLarnon J. G. (2003). Amyloid β peptide-induced corpus callosum damage and glial activation *in vivo*. *Neuroreport* 14 1429–1433. 10.1097/00001756-200308060-00005 12960758

[B35] Jawhar S. Trawicka A. Jenneckens C. Bayer T. A. Wirths O. (2012). Motor deficits, neuron loss, and reduced anxiety coinciding with axonal degeneration and intraneuronal Aβ aggregation in the 5XFAD mouse model of Alzheimer’s disease. *Neurobiol. Aging* 33 196.e29–196.e40. 10.1016/j.neurobiolaging.2010.05.027 20619937

[B36] Jeng L. J. B. Balice-Gordon R. J. Messing A. Fischbeck K. H. Scherer S. S. (2006). The effects of a dominant connexin32 mutant in myelinating Schwann cells. *Mol. Cell. Neurosci.* 32 283–298. 10.1016/j.mcn.2006.05.001 16790356

[B37] Kamphuis W. Middeldorp J. Kooijman L. Sluijs J. A. Kooi E. J. Moeton M. (2014). Glial fibrillary acidic protein isoform expression in plaque related astrogliosis in Alzheimer’s disease. *Neurobiol. Aging* 35 492–510. 10.1016/j.neurobiolaging.2013.09.035 24269023

[B38] Kaya I. Jennische E. Lange S. Tarik Baykal A. Malmberg P. Fletcher J. S. (2020). Brain region-specific amyloid plaque-associated myelin lipid loss, APOE deposition and disruption of the myelin sheath in familial Alzheimer’s disease mice. *J. Neurochem.* 154 7–10. 10.1111/jnc.14999 32141089

[B39] Kielian T. (2008). Glial connexins and gap junctions in CNS inflammation and disease. *J. Neurochem.* 106 1000–1016. 10.1111/j.1471-4159.2008.05405.x 18410504 PMC2648839

[B40] Kleopa K. A. Orthmann J. L. Enriquez A. Paul D. L. Scherer S. S. (2004). Unique distributions of the gap junction proteins connexin29, connexin32, and connexin47 in oligodendrocytes. *Glia* 47 346–357. 10.1002/glia.20043 15293232

[B41] Kobayakawa Y. Masaki K. Yamasaki R. Shiraishi W. Hayashida S. Hayashi S. (2018). Downregulation of neuronal and dendritic Connexin36-made electrical synapses without glutamatergic axon terminals in spinal anterior horn cells from the early stage of amyotrophic lateral sclerosis. *Front. Neurosci.* 12:894. 10.3389/fnins.2018.00894 30546295 PMC6279874

[B42] Koulakoff A. Mei X. Orellana J. A. Sáez J. C. Giaume C. (2012). Glial connexin expression and function in the context of Alzheimer’s disease. *Biochim. Biophys. Acta Biomembr.* 1818 2048–2057. 10.1016/j.bbamem.2011.10.001 22008509

[B43] Kuchibhotla K. V. Goldman S. T. Lattarulo C. R. Wu H. Y. Hyman B. T. Bacskai B. J. (2008). Aβ plaques lead to aberrant regulation of calcium homeostasis *in vivo* resulting in structural and functional disruption of neuronal networks. *Neuron* 35 492–510. 10.1016/j.neuron.2008.06.008 18667150 PMC2578820

[B44] Lee J. T. Xu J. Lee J. M. Ku G. Han X. Yang D. I. (2004). Amyloid-β peptide induces oligodendrocyte death by activating the neutral sphingomyelinase-ceramide pathway. *J. Cell Biol.* 164 123–131. 10.1083/jcb.200307017 14709545 PMC2171973

[B45] Lewis J. Dickson D. W. Lin W. L. Chisholm L. Corral A. Jones G. (2001). Enhanced neurofibrillary degeneration in transgenic mice expressing mutant tau and APP. *Science* 293 1487–1491. 10.1126/science.1058189 11520987

[B46] Lin M. T. Beal M. F. (2006). Mitochondrial dysfunction and oxidative stress in neurodegenerative diseases. *Nature* 443 787–795. 10.1038/nature05292 17051205

[B47] Lutz S. E. Zhao Y. Gulinello M. Lee S. C. Raine C. S. Brosnan C. F. (2009). Deletion of astrocyte connexins 43 and 30 leads to a dysmyelinating phenotype and hippocampal CA1 vacuolation. *J. Neurosci.* 29 7743–7752. 10.1523/JNEUROSCI.0341-09.2009 19535586 PMC2737812

[B48] Maarouf C. L. Kokjohn T. A. Whiteside C. M. Macias M. P. Kalback W. M. Sabbagh M. N. (2013). Molecular Differences and Similarities between Alzheimer’s Disease and the 5XFAD Transgenic Mouse Model of Amyloidosis. *Biochem. Insights* 6:bci-s13025. 10.4137/bci.s13025 25210460 PMC4154482

[B49] Magnotti L. M. Goodenough D. A. Paul D. L. (2011). Functional heterotypic interactions between astrocyte and oligodendrocyte connexins. *Glia* 59 26–34. 10.1002/glia.21073 21046554 PMC3022368

[B50] Markoullis K. Sargiannidou I. Gardner C. Hadjisavvas A. Reynolds R. Kleopa K. A. (2012a). Disruption of oligodendrocyte gap junctions in experimental autoimmune encephalomyelitis. *Glia* 60 1053–1066. 10.1002/glia.22334 22461072

[B51] Markoullis K. Sargiannidou I. Schiza N. Hadjisavvas A. Roncaroli F. Reynolds R. (2012b). Gap junction pathology in multiple sclerosis lesions and normal-appearing white matter. *Acta Neuropathol.* 123 873–886. 10.1007/s00401-012-0978-4 22484441

[B52] Markoullis K. Sargiannidou I. Schiza N. Roncaroli F. Reynolds R. Kleopa K. A. (2014). Oligodendrocyte gap junction loss and disconnection from reactive astrocytes in multiple sclerosis gray matter. *J. Neuropathol. Exp. Neurol.* 73 865–879. 10.1097/NEN.0000000000000106 25101702

[B53] Masaki K. (2013). Connexin pathology in acute multiple sclerosis, Baló’s disease and neuromyelitis optica. *Clin. Exp. Neuroimmunol.* 4 36–44. 10.1111/cen3.12062

[B54] Masaki K. Suzuki S. O. Matsushita T. Matsuoka T. Imamura S. Yamasaki R. (2013). Connexin 43 astrocytopathy linked to rapidly progressive multiple sclerosis and neuromyelitis optica. *PLoS One* 8:e72919. 10.1371/journal.pone.0072919 23991165 PMC3749992

[B55] Masaki K. Suzuki S. O. Matsushita T. Yonekawa T. Matsuoka T. Isobe N. (2012). Extensive loss of connexins in Baló’s disease: evidence for an auto-antibody-independent astrocytopathy via impaired astrocyte-oligodendrocyte/myelin interaction. *Acta Neuropathol.* 123 887–900. 10.1007/s00401-012-0972-x 22438105

[B56] May D. Tress O. Seifert G. Willecke K. (2013). Connexin47 protein phosphorylation and stability in oligodendrocytes depend on expression of connexin43 protein in astrocytes. *J. Neurosci.* 33 7985–7996. 10.1523/JNEUROSCI.5874-12.2013 23637189 PMC6618970

[B57] Mei X. Ezan P. Giaume C. Koulakoff A. (2010). Astroglial connexin immunoreactivity is specifically altered at β-amyloid plaques in β-amyloid precursor protein/presenilin1 mice. *Neuroscience* 171 92–105. 10.1016/j.neuroscience.2010.08.001 20813165

[B58] Modi K. K. Roy A. Brahmachari S. Rangasamy S. B. Pahan K. (2015). Cinnamon and its metabolite sodium benzoate attenuate the activation of p21rac and protect memory and learning in an animal model of Alzheimer’s disease. *PLoS One* 10:e0130398. 10.1371/journal.pone.0130398 26102198 PMC4478015

[B59] Nagy J. I. Ionescu A. V. Lynn B. D. Rash J. E. (2003). Connexin29 and connexin32 at oligodendrocyte and astrocyte gap junctions and in myelin of the mouse central nervous system. *J. Comp. Neurol.* 464 356–370. 10.1002/cne.10797 12900929 PMC1859856

[B60] Nagy J. I. Li W. Hertzberg E. L. Marotta C. A. (1996). Elevated connexin43 immunoreactivity at sites of amyloid plaques in Alzheimer’s disease. *Brain Res.* 717 173–178. 10.1016/0006-8993(95)01526-4 8738268

[B61] Nathan C. Calingasan N. Nezezon J. Ding A. Lucia M. S. La Perle K. (2005). Protection from Alzheimer’s-like disease in the mouse by genetic ablation of inducible nitric oxide synthase. *J. Exp. Med.* 202 1163–1116. 10.1084/jem.20051529 16260491 PMC2213235

[B62] Navarro V. Sanchez-Mejias E. Jimenez S. Muñoz-Castro C. Sanchez-Varo R. Davila J. C. (2018). Microglia in Alzheimer’s disease: activated, dysfunctional or degenerative. *Front. Aging Neurosci.* 10:140. 10.3389/fnagi.2018.00140 29867449 PMC5958192

[B63] Oakley H. Cole S. L. Logan S. Maus E. Shao P. Craft J. (2006). Intraneuronal beta-amyloid aggregates, neurodegeneration, and neuron loss in transgenic mice with five familial Alzheimer’s disease mutations: potential factors in amyloid plaque formation. *J. Neurosci.* 26 10129–10140. 10.1523/JNEUROSCI.1202-06.2006 17021169 PMC6674618

[B64] Oddo S. Caccamo A. Shepherd J. D. Murphy M. P. Golde T. E. Kayed R. (2003). Triple-transgenic model of Alzheimer’s Disease with plaques and tangles: intracellular Aβ and synaptic dysfunction. *Neuron* 39 409–421. 10.1016/S0896-6273(03)00434-3 12895417

[B65] Odermatt B. Wellershaus K. Wallraff A. Seifert G. Degen J. Euwens C. (2003). Connexin 47 (Cx47)-Deficient Mice with Enhanced Green Fluorescent Protein Reporter Gene Reveal Predominant Oligodendrocytic Expression of Cx47 and Display Vacuolized Myelin in the CNS. *J. Neurosci.* 23 4549–4559. 10.1523/jneurosci.23-11-04549.2003 12805295 PMC6740816

[B66] Ohno M. Chang L. Tseng W. Oakley H. Citron M. Klein W. L. (2006). Temporal memory deficits in Alzheimer’s mouse models: rescue by genetic deletion of BACE1. *Eur. J. Neurosci.* 23 251–260. 10.1111/j.1460-9568.2005.04551.x 16420434

[B67] Orellana J. A. Froger N. Ezan P. Jiang J. X. Bennett M. V. L. Naus C. C. (2011a). ATP and glutamate released via astroglial connexin 43 hemichannels mediate neuronal death through activation of pannexin 1 hemichannels. *J. Neurochem.* 118 826–840. 10.1111/j.1471-4159.2011.07210.x 21294731 PMC3108012

[B68] Orellana J. A. Sáez P. J. Shoji K. F. Schalper K. A. Palacios-Prado N. Velarde V. (2009). Modulation of brain hemichannels and gap junction channels by pro-inflammatory agents and their possible role in neurodegeneration. *Antioxid. Redox Signal.* 11 369–399. 10.1089/ars.2008.2130 18816186 PMC2713807

[B69] Orellana J. A. Shoji K. F. Abudara V. Ezan P. Amigou E. Saez P. J. (2011b). Amyloid -induced death in neurons involves glial and neuronal hemichannels. *J. Neurosci.* 31 4962–4977. 10.1523/JNEUROSCI.6417-10.2011 21451035 PMC6622997

[B70] Orthmann-Murphy J. L. Freidin M. Fischer E. Scherer S. S. Abrams C. K. (2007). Two distinct heterotypic channels mediate gap junction coupling between astrocyte and oligodendrocyte connexins. *J. Neurosci.* 27 13949–13957. 10.1523/JNEUROSCI.3395-07.2007 18094232 PMC6673504

[B71] Papaneophytou C. Georgiou E. Kleopa K. A. (2019). The role of oligodendrocyte gap junctions in neuroinflammation. *Channels* 13 247–263. 10.1080/19336950.2019.1631107 31232168 PMC6602578

[B72] Parihar M. S. Brewer G. J. (2010). Amyloid-β as a modulator of synaptic plasticity. *J. Alzheimers Dis.* 22 741–763. 10.3233/JAD-2010-101020 20847424 PMC3079354

[B73] Paznekas W. A. Boyadjiev S. A. Shapiro R. E. Daniels O. Wollnik B. Keegan C. E. (2003). Connexin 43 (GJA1) mutations cause the pleiotropic phenotype of oculodentodigital dysplasia. *Am. J. Hum. Genet.* 72 408–418. 10.1086/346090 12457340 PMC379233

[B74] Pelegrin P. Surprenant A. (2006). Pannexin-1 mediates large pore formation and interleukin-1β release by the ATP-gated P2X7 receptor. *EMBO J.* 25 5071–5582. 10.1038/sj.emboj.7601378 17036048 PMC1630421

[B75] Pelegrin P. Surprenant A. (2009). The P2X7 receptor - Pannexin connection to dye uptake and IL-1β release. *Purinergic Signal.* 5 129–137. 10.1007/s11302-009-9141-7 19212823 PMC2686830

[B76] Perez-Nievas B. G. Serrano-Pozo A. (2018). Deciphering the astrocyte reaction in Alzheimer’s disease. *Front. Aging Neurosci.* 10:114. 10.3389/fnagi.2018.00114 29922147 PMC5996928

[B77] Radde R. Bolmont T. Kaeser S. A. Coomaraswamy J. Lindau D. Stoltze L. (2006). Aβ42-driven cerebral amyloidosis in transgenic mice reveals early and robust pathology. *EMBO Rep.* 7 940–946. 10.1038/sj.embor.7400784 16906128 PMC1559665

[B78] Reiss A. B. Arain H. A. Stecker M. M. Siegart N. M. Kasselman L. J. (2018). Amyloid toxicity in Alzheimer’s disease. *Rev. Neurosci.* 29 613–627. 10.1515/revneuro-2017-0063 29447116

[B79] Retamal M. A. Froger N. Palacios-Prado N. Ezan P. Saez P. J. Saez J. C. (2007). Cx43 hemichannels and gap junction channels in astrocytes are regulated oppositely by proinflammatory cytokines released from activated microglia. *J. Neurosci.* 27 13781–13792. 10.1523/JNEUROSCI.2042-07.2007 18077690 PMC6673621

[B80] Roher A. E. Weiss N. Kokjohn T. A. Kuo Y. M. Kalback W. Anthony J. (2002). Increased Aβ peptides and reduced cholesterol and myelin proteins characterize white matter degeneration in Alzheimer’s disease. *Biochemistry* 41 11080–11090. 10.1021/bi026173d 12220172

[B81] Roscoe W. A. Kidder G. M. Karlik S. J. (2007a). Experimental allergic encephalomyelitis in connexin 43-heterozygous mice. *Cell Commun. Adhes.* 14 57–73. 10.1080/15419060701459569 17668350

[B82] Roscoe W. A. Messersmith E. Meyer-Franke A. Wipke B. Karlik S. J. (2007b). Connexin 43 gap junction proteins are up-regulated in remyelinating spinal cord. *J. Neurosci. Res.* 85 945–953. 10.1002/jnr.21194 17279545

[B83] Rufer M. Wirth S. B. Hofer A. Dermietzel R. Pastor A. Kettenmann H. (1996). Regulation of connexin-43, GFAP, and FGF-2 is not accompanied by changes in astroglial coupling in MPTP-lesioned, FGF-2-treated parkinsonian mice. *J. Neurosci. Res.* 46 606–617. 10.1002/(sici)1097-4547(19961201)46:5<606::aid-jnr9>3.0.co;2-n 8951672

[B84] Sargiannidou I. Vavlitou N. Markoullis K. Kyriacou K. Scherer S. S. Kleopa K. A. (2009). Axonal degeneration in mouse models of CMT1X neuropathy. *Glia* 57:s118. 10.1097/NEN.0b013e3181efa658 PMC3034224 20720503

[B85] Scemes E. Giaume C. (2006). Astrocyte calcium waves: What they are and what they do. *Glia* 54 716–725. 10.1002/glia.20374 17006900 PMC2605018

[B86] Sjöbeck M. Haglund M. Englund E. (2005). Decreasing myelin density reflected increasing white matter pathology in azheimer’s disease - A neuropathological study. *Int. J. Geriatr. Psychiatry* 20 919–926. 10.1002/gps.1384 16163742

[B87] Skaper S. D. (2012). Alzheimer’s disease and amyloid: Culprit or coincidence? *Int. Rev. Neurobiol.* 102 277–316. 10.1016/B978-0-12-386986-9.00011-9 22748834

[B88] Smith P. K. Krohn R. I. Hermanson G. T. Mallia A. K. Gartner F. H. Provenzano M. D. (1985). Measurement of protein using bicinchoninic acid. *Anal. Biochem.* 150 76–85. 10.1016/0003-2697(85)90442-7 3843705

[B89] Sturchler-Pierrat C. Abramowski D. Duke M. Wiederhold K. H. Mistl C. Rothacher S. (1997). Two amyloid precursor protein transgenic mouse models with Alzheimer disease-like pathology. *Proc. Natl. Acad. Sci. U.S.A.* 94 13287–13292. 10.1073/pnas.94.24.13287 9371838 PMC24301

[B90] Sutor B. Schmolke C. Teubner B. Schirmer C. Willecke K. (2000). Myelination defects and neuronal hyperexcitability in the neocortex of Connexin 32-deficient mice. *Cereb. Cortex* 23 4549–4559. 10.1093/cercor/10.7.684 10906315

[B91] Takeuchi H. Suzumura A. (2014). Gap junctions and hemichannels composed of connexins: potential therapeutic targets for neurodegenerative diseases. *Front. Cell. Neurosci.* 8:189. 10.3389/fncel.2014.00189 25228858 PMC4151093

[B92] Talantova M. Sanz-Blasco S. Zhang X. Xia P. Akhtar M. W. Okamoto S. I. (2013). Aβ induces astrocytic glutamate release, extrasynaptic NMDA receptor activation, and synaptic loss. *Proc. Natl. Acad. Sci. U.S.A.* 110 E2518–E2527. 10.1073/pnas.1306832110 23776240 PMC3704025

[B93] Tress O. Maglione M. May D. Pivneva T. Richter N. Seyfarth J. (2012). Panglial gap junctional communication is essential for maintenance of myelin in the CNS. *J. Neurosci.* 32 7499–7518. 10.1523/JNEUROSCI.0392-12.2012 22649229 PMC6703577

[B94] Uhlenberg B. Schuelke M. Rüschendorf F. Ruf N. Kaindl A. M. Henneke M. (2004). Mutations in the gene encoding gap junction protein α12 (Connexin 46.6) cause Pelizaeus-Merbacher-like disease. *Am. J. Hum. Genet.* 75 251–260. 10.1086/422763 15192806 PMC1216059

[B95] Watanabe M. Masaki K. Yamasaki R. Kawanokuchi J. Takeuchi H. Matsushita T. (2016). Th1 cells downregulate connexin 43 gap junctions in astrocytes via microglial activation. *Sci. Rep.* 6:38387. 10.1038/srep38387 27929069 PMC5143974

[B96] Wu C. Y. Hsieh H. L. Jou M. J. Yang C. M. (2004). Involvement of p42/p44 MAPK, p38 MAPK, JNK and nuclear factor-kappa B in interleukin-1β-induced matrix metalloproteinase-9 expression in rat brain astrocytes. *J. Neurochem.* 90 1477–1488. 10.1111/j.1471-4159.2004.02682.x 15341531

[B97] Wu D. Tang X. Gu L. H. Li X. L. Qi X. Y. Bai F. (2018). LINGO-1 antibody ameliorates myelin impairment and spatial memory deficits in the early stage of 5XFAD mice. *CNS Neurosci. Ther.* 24 381–393. 10.1111/cns.12809 29427384 PMC6489849

[B98] Xiao N. A. Zhang J. Zhou M. Wei Z. Wu X. L. Dai X. M. (2015). Reduction of glucose metabolism in olfactory bulb is an earlier Alzheimer’s disease-related biomarker in 5XFAD mice. *Chin. Med. J.* 128 2220–2227. 10.4103/0366-6999.162507 26265617 PMC4717990

[B99] Xing L. Y. Yang T. Cui S. Sen Chen G. (2019). Connexin hemichannels in astrocytes: role in CNS disorders. *Front. Mol. Neurosci.* 12:23. 10.3389/fnmol.2019.00023 30787868 PMC6372977

[B100] Xu C. Y. Zhang W. S. Zhang H. Cao Y. Zhou H. Y. (2019). The Role of Connexin-43 in the inflammatory process: a new potential therapy to influence Keratitis. *J. Ophthalmol.* 2019:9312827. 10.1155/2019/9312827 30805212 PMC6360563

[B101] Yamasaki R. (2018). Connexins in health and disease. *Clin. Exp. Neuroimmunol.* 9 30–36. 10.1111/cen3.12433

[B102] Yan J. Thomson J. K. Zhao W. Wu X. Gao X. DeMarco D. (2018). The stress kinase JNK regulates gap junction Cx43 gene expression and promotes atrial fibrillation in the aged heart. *J. Mol. Cell. Cardiol.* 114 105–115. 10.1016/j.yjmcc.2017.11.006 29146153 PMC5800987

[B103] Yi C. Mei X. Ezan P. Mato S. Matias I. Giaume C. (2016). Astroglial connexin43 contributes to neuronal suffering in a mouse model of Alzheimer’s disease. *Cell Death Differ.* 23 1691–1701. 10.1038/cdd.2016.63 27391799 PMC5041199

[B104] Yool D. A. Edgar J. M. Montague P. Malcolm S. (2000). The proteolipid protein gene and myelin disorders in man and animal models. *Hum. Mol. Genet.* 76 813–825. 10.1093/hmg/9.6.987 10767322

