# Supplementary material for: Altered Expression of Glial Gap Junction Proteins Cx43, Cx30, and Cx47 in the 5XFAD Model of Alzheimer’s Disease
Source: Front Neurosci. 2020 Oct 7;14:582934. doi: 10.3389/fnins.2020.582934 (PMC7575794; doi:10.3389/fnins.2020.582934)
Supplement: Supplementary file 1 [file Data_Sheet_1.DOCX]

***Supplementary material***

#
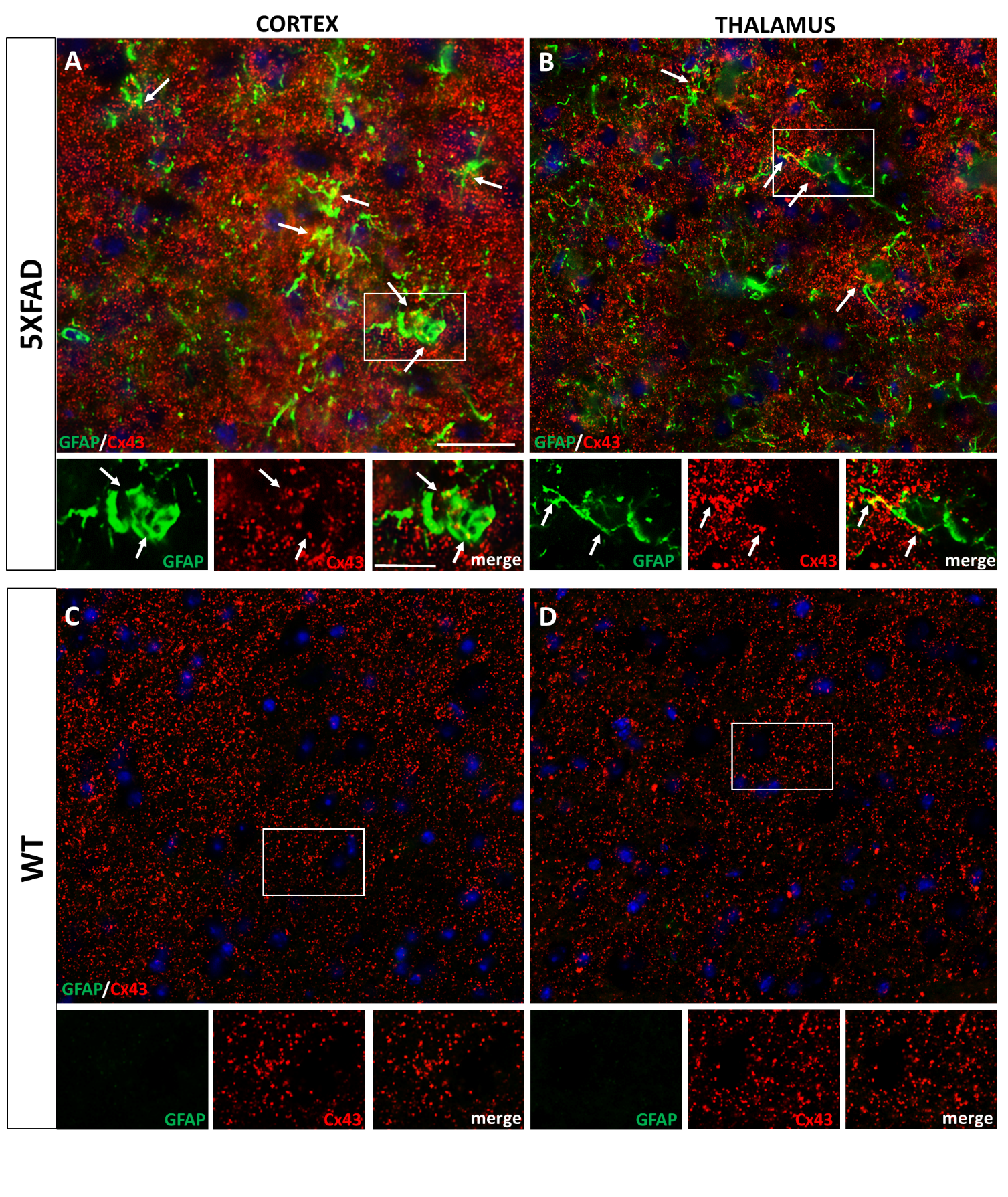
**Supplementary Figures**

**Supplementary Figure 1.** Localization of Cx43 plaques to GFAP immunoreactive processes (white arrows) in the cortex (A) and thalamus (B) of 9-month-old 5XFAD mice. Cell nuclei were counterstained with DAPI (blue).5XFAD mice showed increased immunoreactivity of Cx43 in the cortex and thalamus compared to WT mice (C, D). Cx43 plaques which do not colocalize with GFAP could be either expressed by other astrocytes which are not GFAP positive or by microglia. Magnification in A-D, x20. Scale bars = 50 μm; 25 μm in higher magnification insets.


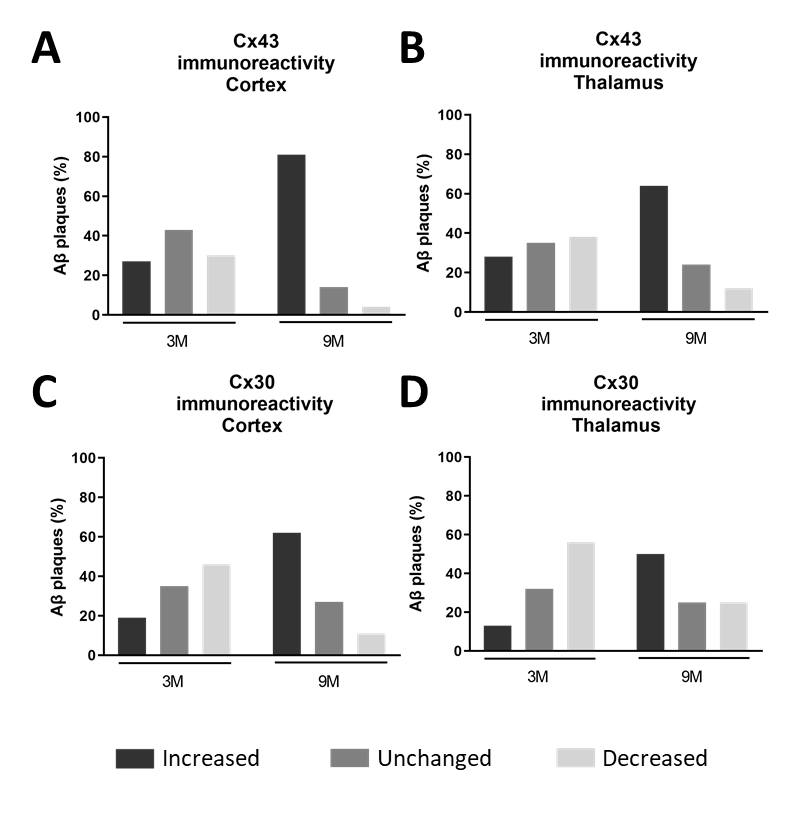


**Supplementary Figure 2.** Aβ plaque percentage with either increased, unchanged or decreased Cx43 and Cx30 immunoreactivity in the immediate vicinity of Aβ plaques, compared to areas away from plaques. **(A, B)** In the cortex and thalamus of 3-month-old 5XFAD mice, the percentages of Aβ plaques that showed increased, unchanged and decreased Cx43 immunoreactivity had no major differences. However, in 9-month-old mice, the majority of Aβ plaques showed increased Cx43 immunoreactivity in their microenvironment compared to areas away from Aβ plaques, in both cortex and thalamus. **(C, D)** In the cortex and thalamus of 3-month-old 5XFAD mice, the majority of Aβ plaques showed decreased Cx30 immunoreactivity, compared to areas away from Aβ plaques. However, in 9-month-old 5XFAD mice, the majority of Aβ plaques showed increased Cx30 immunoreactivity, compared to areas away from Aβ plaques, in both brain regions.


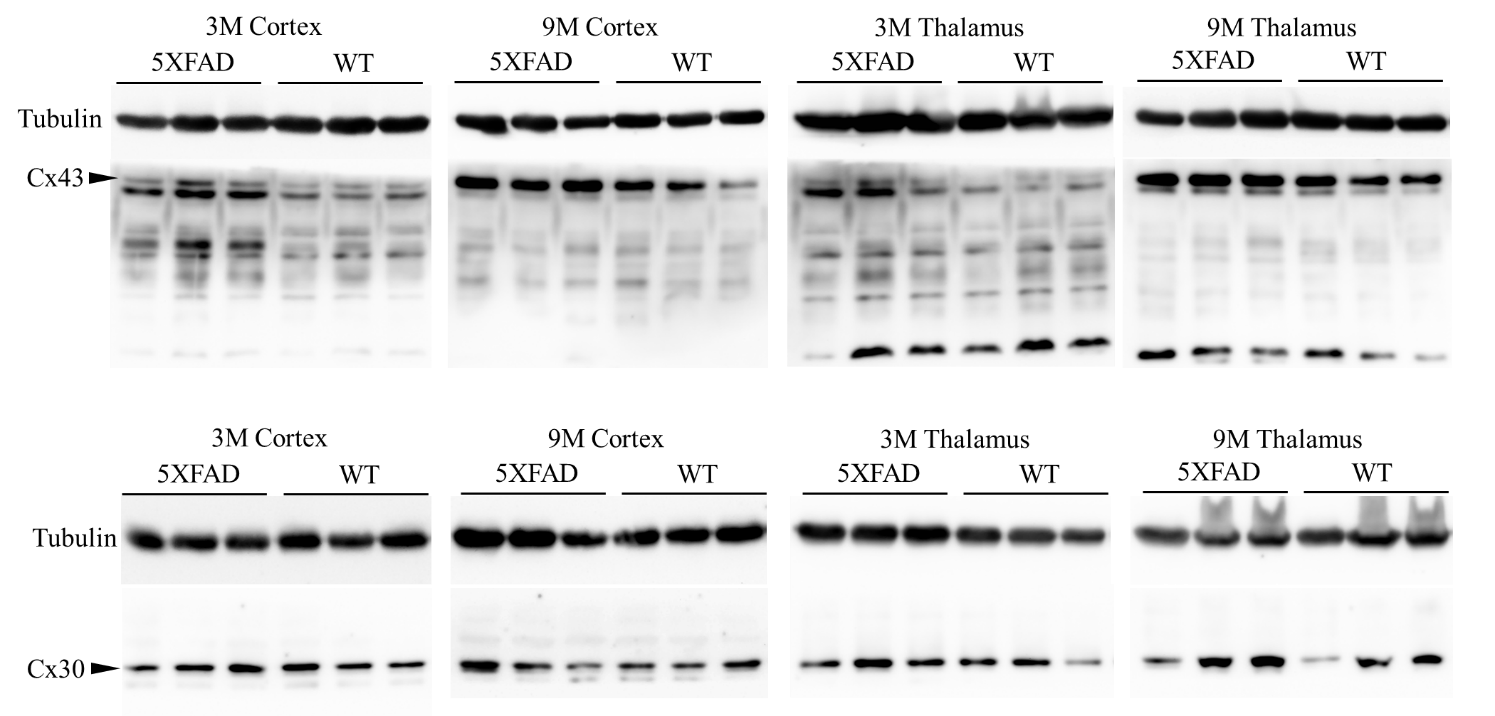


**Supplementary Figure 3.** Immunoblots of original gels showing Cx43 and Cx30 in the cortex and thalamus of 5XFAD and WT mice.


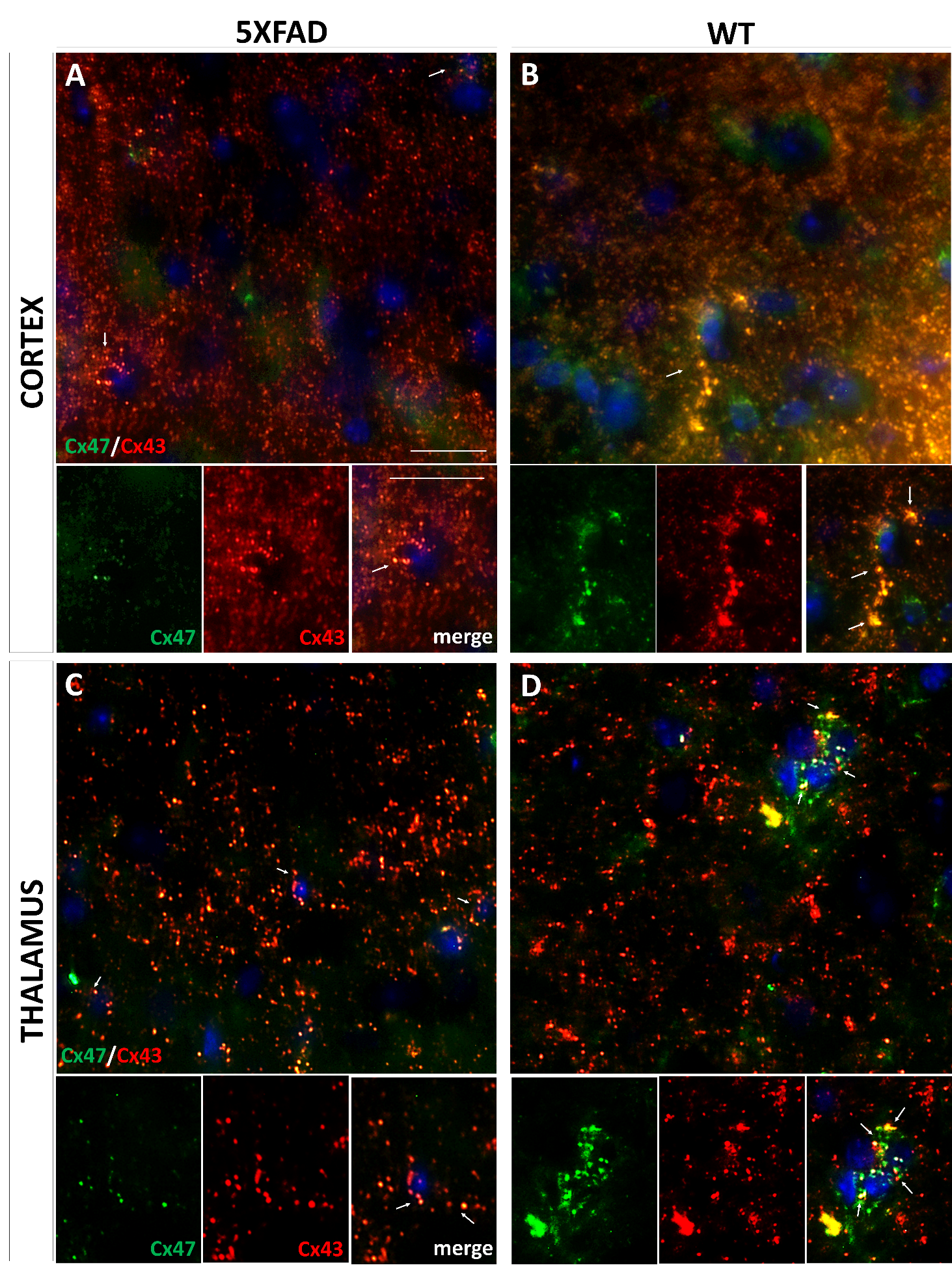


**Supplementary Figure 4.** Loss of Cx47/Cx43 gap junctions (GJs) in 9-month-old 5XFAD mice in the cortex and thalamus. Double immunofluorescence staining for Cx47 and Cx43 showed reduced Cx47 GJ plaques with less colocalization with Cx43 plaques which are diffusely increased in 5XFAD compared to WT mice. Cell nuclei were counterstained with DAPI (blue). Higher magnification insets confirm decreased colocalization of Cx47 and Cx43 GJs (white arrows) in 5XFAD (A, C) compared to WT (B,D) mice, confirming loss of A/O GJs in the setting of astrogliosis and increased Cx43 expression. Scale bar = 25μm.


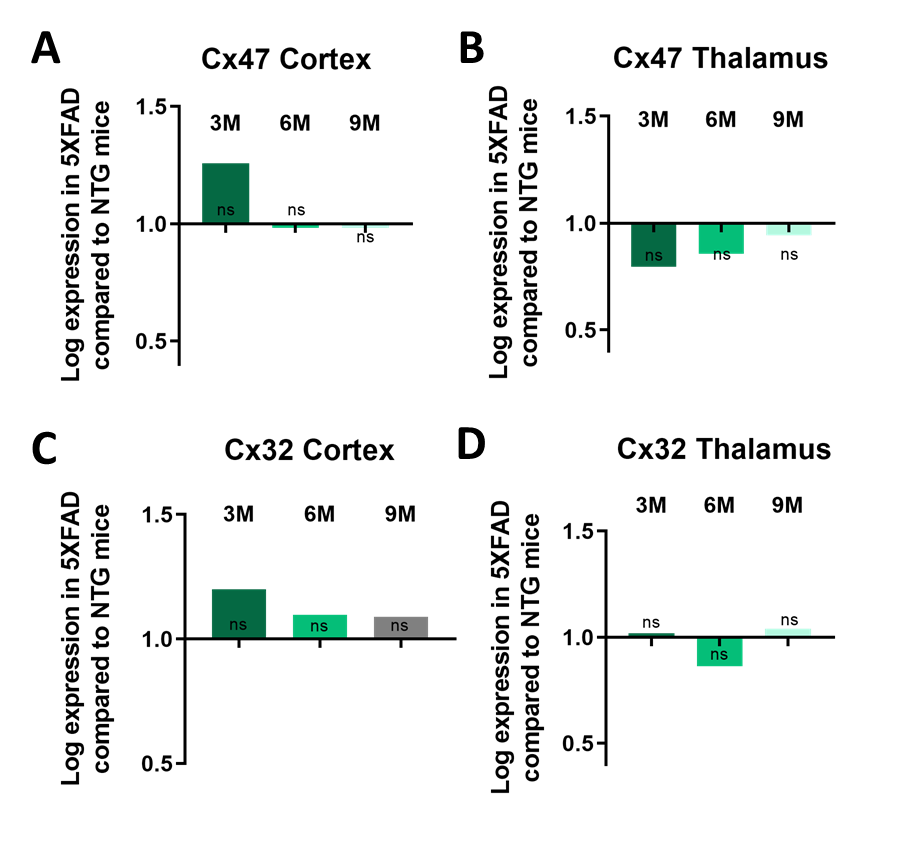


**Supplementary Figure 5.** Real-time PCR analysis of Cx47 and Cx32 mRNA levels in the brain of 5XFAD mice. There was no significant difference in the expression levels of Cx47 and Cx32 between 5XFAD and WT mice in the cortex and thalamus. The statistical analysis for mRNA was performed by one-way ANOVA followed by Kruskal-Wallis multiple comparisons test (5XFAD mice/age (n=6), WT mice/age (n=6)). ns= non-significant.


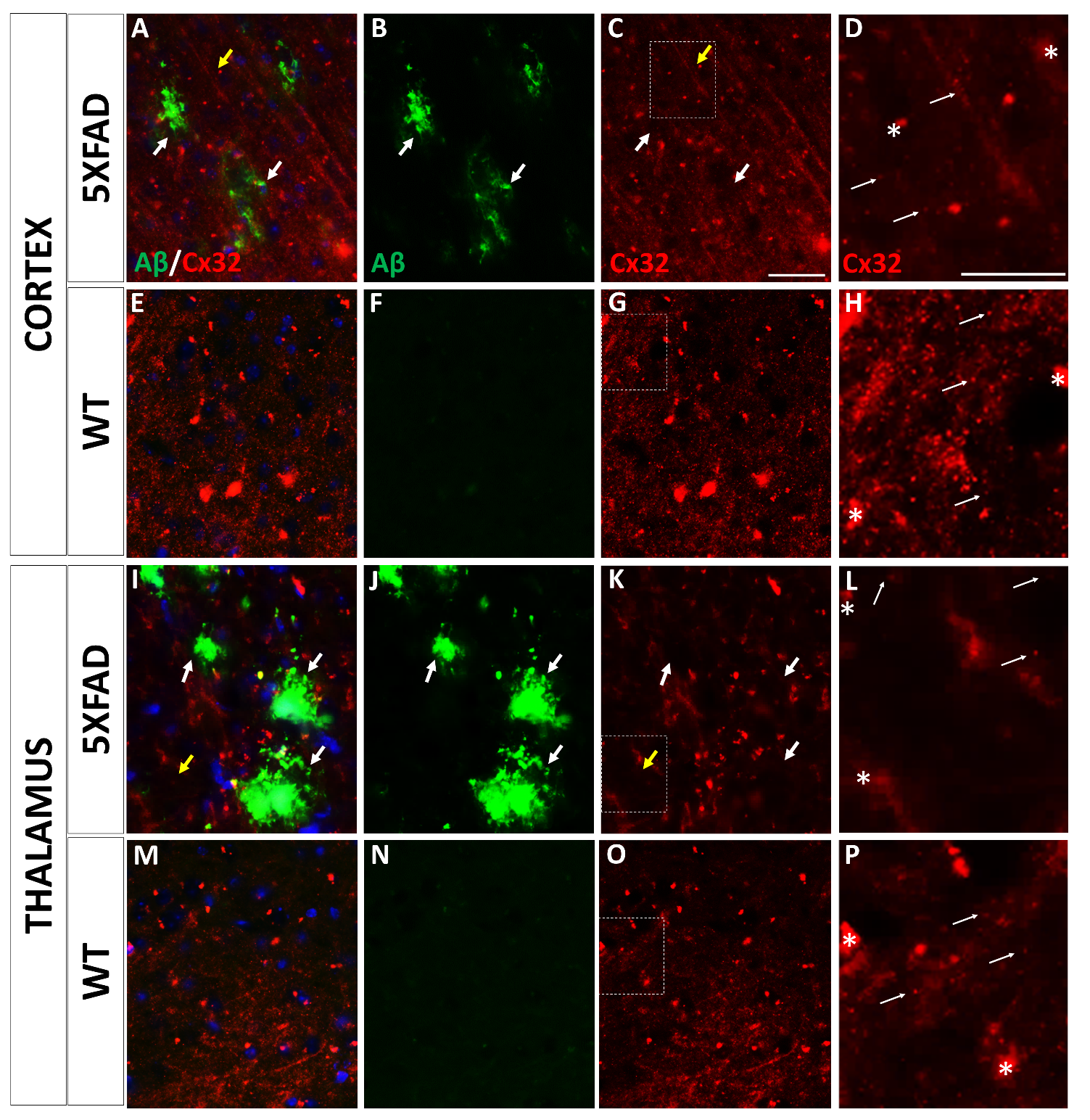


**Supplementary Figure 6.** Cx32 distribution in the cortex and thalamus of 9-month-old 5XFAD and WT mice. Double immunofluorescence staining of Aβ/Cx32 indicates that the specific diffuse immunoreactivity of Cx32 GJ plaques in 5XFAD is reduced compared to WT mice in both brain areas. Cell nuclei were counterstained with DAPI (blue). Cx32 appears disrupted (A-C, I-K) both within (thick white arrows) and in areas outside Aβ plaques (yellow arrows). In higher magnification insets (D, H, L, P) the thin white arrows indicate the specific immunoreactivity of Cx32 GJ plaques, while the asterisks indicate scattered intense signals which are artefacts in both 5XFAD and WT images. Scale bar in A-C, E-G, I-K, M-O = 50 μm; scale bar in higher magnification insets D, H, L, P =25 μm.


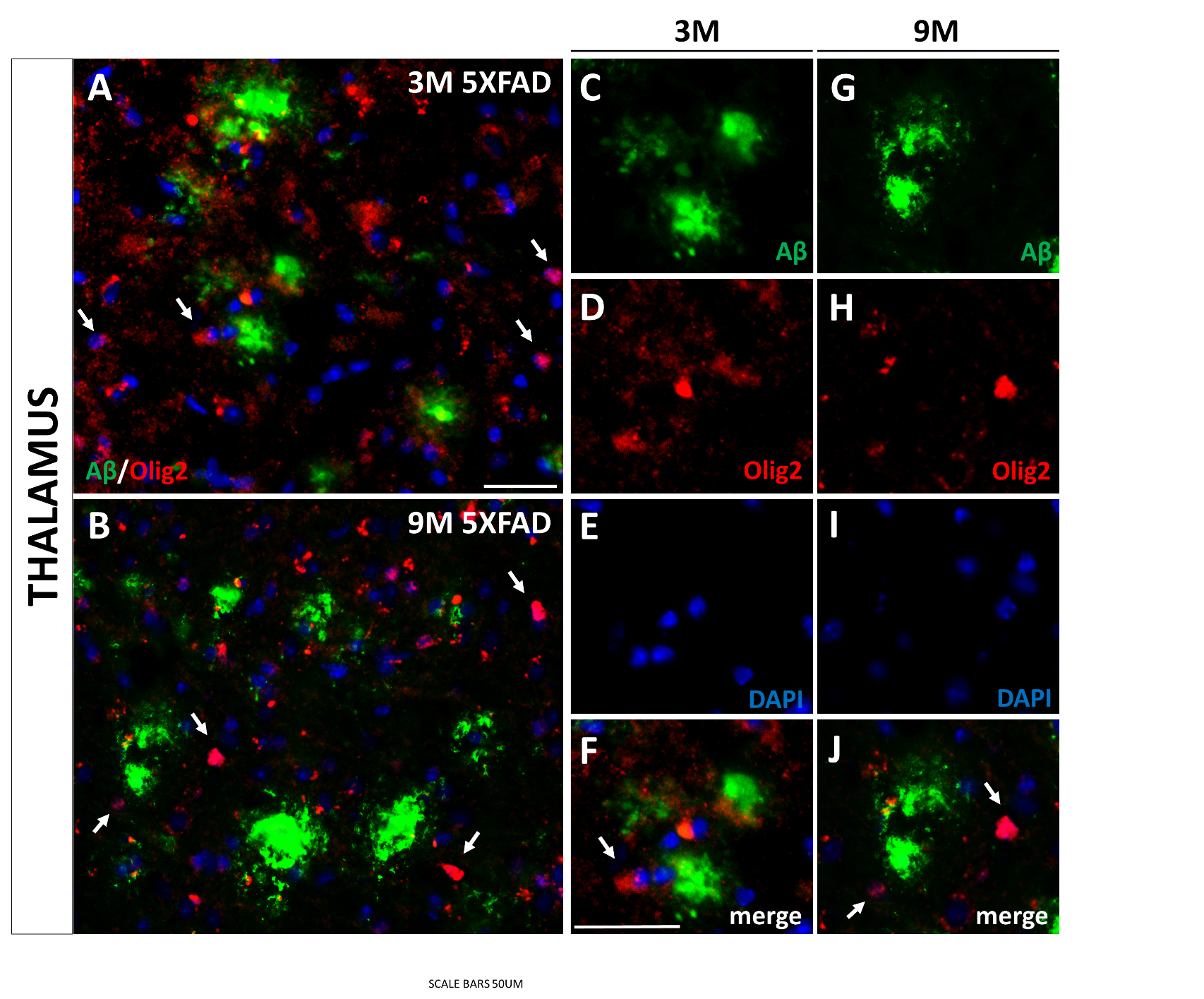


**Supplementary Figure 7.**  Lack of Olig2^+^ cell clusters in 5XFAD mice. Double immunofluorescence staining in the thalamus of 3-month (A) and 9-month-old (B) 5XFAD mice with Aβ/Olig2 (green/red). Cell nuclei are counterstained with DAPI (blue). Olig2^+^ cells (white arrows) were not found in clusters around Aβ plaques but rather diffusely distributed. Higher magnification insets from panel A (C-F) and from panel B (G-J). Scale bar = 50 μm
